# MSO: A Modified Snake Optimizer for Engineering Applications

**DOI:** 10.3390/biomimetics11020137

**Published:** 2026-02-12

**Authors:** Hongxi Wang, Likun Hu

**Affiliations:** School of Electrical Engineering, Guangxi University, Nanning 530004, China; 2312301056@st.gxu.edu.cn

**Keywords:** snake optimizer, dual mapping, opposition-based learning, RIME, UAV path planning, engineering optimization problems

## Abstract

Many complex engineering problems can be formulated as mathematical optimization tasks, for which bio-inspired metaheuristic algorithms have demonstrated outstanding effectiveness. Drawing inspiration from snake behavior, the Snake Optimizer (SO) algorithm provides a promising framework but suffers from random population initialization, insufficient global search capability, and slow convergence. To address these drawbacks, the study proposes a Modified Snake Optimizer (MSO) that integrates three key strategies: a dual mapping strategy based on Latin hypercube sampling and logistic mapping for population initialization; an opposition-based learning mechanism with scaling factors for exploration; and integration of the soft-rime search strategy from RIME optimization during exploitation. The performance of MSO was benchmarked against nine representative algorithms using the CEC2017 and further validated on three engineering application problems—pressure vessel, tension/compression spring, and hydrostatic thrust bearing design, and two UAV path planning scenarios. Experimental results show that MSO achieves faster convergence speed, stronger robustness and greater stability, effectively extending the biomimetic principles of the original SO and confirming its superiority for solving optimization problems.

## 1. Introduction

With the rapid development of technology and society, academic research is increasingly confronted with complex optimization problems across various fields, such as engineering, mathematics, and physics—particularly in emerging domains such as UAV path planning [1]. These optimization challenges require identifying the best possible solutions under some specific conditions. As the scale and complexity of real-world optimization problems continue to increase, traditional deterministic mathematical optimization methods, such as gradient descent, reveal inherent limitations. These include the need for an explicitly defined search space, strong dependence on the continuity of the objective function, and frequent entrapment in local optima. Research on metaheuristic optimization algorithms has enriched the theoretical foundations of optimization techniques, enhanced understanding of their performance and convergence characteristics, and provided effective solutions for complex engineering applications. A crucial advantage of metaheuristic algorithms lies in their non-derivative and non-gradient nature, which allows them to be flexibly applied to complex optimization problems and search more intuitively for optimal solutions within the search space. These algorithms are particularly well-suited for solving non-convex, nonlinear, and continuous optimization problems where classic mathematical techniques are usually unable to achieve satisfactory results. Metaheuristic algorithms have demonstrated remarkable effectiveness across a wide range of domains, especially where deterministic approaches are inadequate. Their main advantages include simplicity, flexibility, and independence from problem-specific properties [2]. For instance, in UAV path planning, metaheuristic algorithms can efficiently address multiple objectives and rapidly generate feasible flight trajectories [3]; in wireless sensor network coverage problems, they achieve higher coverage with fewer nodes, thereby enhancing efficiency and reducing deployment costs [4]; and in network routing optimization, they enable more rational clustering strategies that significantly extend network lifetime [5].

Despite remarkable progress in various engineering applications, metaheuristic algorithms continue to face inherent challenges and limitations. As described by the No Free Lunch (NFL) Theorem, every optimization algorithm exhibits strong performance only on specific types of problems, while none can maintain superiority across all possible domains [6]. This limitation has motivated researchers to improve existing algorithms or design new ones better suited to specific classes of optimization problems.

In recent years, numerous metaheuristic algorithms inspired by biological behaviors have been proposed, demonstrating strong capabilities in addressing complex optimization tasks. demonstrating distinct capability in addressing complex optimization problems. For example, the Beluga Whale Optimization (BWO) algorithm mimics the swim, whale-fall, and prey behaviors of beluga whales [7]. It consists of two main phases: an exploration phase, corresponding to the swim behavior, and an exploitation phase, representing the prey behavior. This structure enables BWO to maintain an effective balance between exploration and exploitation, rendering it well-suited for solving complex optimization problems. The Dung Beetle Optimization (DBO) algorithm draws inspiration from the natural behaviors of dung beetles and models their ball-rolling, dancing, foraging, stealing, and reproductive actions [8]. It employs a multi-subpopulation structure in which each subgroup executes distinct search strategies, resulting in outstanding optimization performance. The Crown Porcupine Optimization (CPO) algorithm models four distinct defensive mechanisms—sight, sound, odor, and attack. The first two mechanisms correspond to the exploration phase, while the latter two are associated with the exploitation phase, enabling CPO to simultaneously perform global search and local optimization [9].

Inspired by the foraging and reproductive behaviors of snakes, Hashim and Hussien proposed the Snake Optimizer (SO) in 2022 as a novel swarm-based metaheuristic algorithm [10]. This SO algorithm integrates multiple strategies while requiring relatively few tuning parameters. Despite its promising performance, the SO algorithm still exhibits several limitations, including inefficient exploration, slow convergence speed, random population initialization and a tendency to stagnate in local optima.

To overcome these drawbacks, several researchers have proposed improved variants of SO. Yao et al. proposed the Enhanced Snake Optimizer (ESO) [11], which integrates an opposition-based learning mechanism and a dynamic parameter adjustment strategy to improve search performance. Experimental validation on 23 benchmark functions and four engineering design problems demonstrated that the ESO outperforms 13 other competitive algorithms. Similarly, Alawad et al. introduced the Hybrid Snake Optimizer Algorithm (HSOA) to overcome premature convergence and enhance population diversity of SO, demonstrating competitive performance on the CEC 2014 functions [12].

Hu et al. developed a Multi-strategy Boosted Snake-Inspired Optimizer for Engineering Applications (BEESO) to improve global optimization performance and enhance engineering applicability. BEESO integrates three key strategies—Bi-Directional Search, Modified Evolutionary Population Dynamics, and Elite Opposition-Based Learning [13], to simultaneously increase population diversity and accelerate convergence speed. The evaluation results reveal that BEESO performs better than the original SO algorithm and remains highly competitive compared with other metaheuristic algorithms. Despite this, BEESO still tends to fall into local optima, which limits its optimization performance.

Abdülkadir, Pektas et al. introduced the Snake Optimizer Particle Swarm Optimization (SO–PSO) algorithm [14], which incorporates the velocity vector of Particle Swarm Optimization (PSO) into the exploration phase of SO to enhance search efficiency. Nevertheless, the relatively low quality of the initial population still hinders convergence speed and reduces overall stability.

A comprehensive review of existing studies reveals that the primary limitations of the original Snake Optimizer and its variants lie in poor initial population quality, insufficient global exploration capability, slow convergence, and a tendency to get stuck in local optima—all of which constrain its overall performance and ability to solve engineering application problems. To mitigate these drawbacks, this study proposes a Modified Snake Optimizer (MSO) algorithm. The primary contributions of this work differ from previous studies in the following aspects:

1. A dual-mapping strategy based on Latin hypercube sampling and logistic mapping is proposed for population initialization. This strategy improves the spatial distribution of the initial population and effectively enhances convergence speed.
2. An opposition-based learning mechanism with scaling factors is incorporated during the exploration phase to expand the search range and strengthen the algorithm’s ability to locate global optima.
3. Soft-rime search strategy from RIME is integrated into the exploitation phase, which enhances local search ability and effectively prevents premature convergence.
4. Comprehensive comparative experiments involving nine other algorithms on the CEC 2017 functions, three classical engineering application problems, and two UAV path planning scenarios demonstrate the effectiveness, stability, and superiority of the MSO algorithm in addressing both numerical optimization and real-world engineering problems.

## 2. Snake Optimizer (SO)

Snake Optimizer is a novel metaheuristic algorithm [10], inspired by snakes’ mating, foraging, and competitive behaviors in natural environments. When food is scarce, snakes engage in foraging; under low temperatures with ample food, male and female snakes fight and mate. If food is abundant but temperatures are excessively hot, the snake only moves toward existing food and eats them. The steps of SO are as follows:

### 2.1. Initialization

Similar to other metaheuristic algorithms, the Snake Optimizer algorithm randomly generates initial individuals in the search space:

(1) $$X_{i}=X_{\min}+rand*(X_{\max}-X_{\min})$$

Among these, $X_{i}$ is a D-dimensional vector and represents the position of the ith individual; rand is a randomly generated number between 0 and 1; $X_{\max}$ and $X_{\min}$ denote the upper and lower bounds of the search space, respectively, and are also d-dimensional vectors. The equation is applied independently for each dimension.

### 2.2. Division into Male and Female Subpopulations

After initialization, the population is divided into male and female groups according to the equations:

(2) $$N_{m}=N/2$$

(3) $$N_{f}=N-N_{m}$$

where N is the total number of individuals, and $N_{m}$ and $N_{f}$ represent the number of male and female individuals, respectively.

### 2.3. Calculation of Food Quantity and Temperature

After dividing the population into two subpopulations, calculate the fitness value for each individual. The exploration and exploitation tendencies are determined based on the parameters of Temperature (Temp) and Food quantity (Q):

(4) $$Temp=\exp\left(\frac{-t}{T}\right)$$

(5) $$Q=c_{1}\times \exp\left(\frac{t-T}{T}\right)$$

where t represents the current iteration, T refers to the maximum number of iterations, $c_{1}$ is a constant equal to 0.5.

### 2.4. Exploration Phase (Insufficient Food)

When Q < 0.25, the snake finds food by selecting random positions. The position equations for this phase are calculated as follows:

(6) $$X_{i,m}(t+1)=X_{rand,m}(t)\pm c_{2}\times A_{m}\times \left(\left(X_{\max}-X_{\min}\right)\right.\times rand+X_{\min})$$

(7) $$X_{i,f}(t+1)=X_{rand,f}(t)\pm c_{2}\times A_{f}\times \left(\left(X_{\max}-X_{\min}\right)\right.\times rand+X_{\min})$$

$X_{i,m}$, $X_{i,f}$ refers to the positions of the male and female snakes, respectively; $X_{rand,m}$, $X_{rand,f}$ refers to the selected positions of male and female snakes, respectively; rand is a random number between 0 and 1; $c_{2}$ is a constant equal to 0.05. ± operator is determined by a random number r ∈ [0,1]; if r < 0.5, the negative sign is used, and if r ≥ 0.5, the positive sign is used. $A_{m}$, $A_{f}$ represent the male ability to find the food abilities of the male and female snakes, and can be computed as follows, respectively:

(8) $$A_{m}=\exp\left(\frac{-f_{rand,m}}{f_{i,m}}\right)$$

(9) $$A_{f}=exp\left(\frac{-f_{rand,f}}{f_{i,f}}\right)$$

$f_{rand,m}$ and $f_{rand,f}$ are the fitness of $X_{rand,m}$ and $X_{rand,f}$; $f_{i,m}$ and $f_{i,f}$ are the fitness of ith individuals in male and female groups.

### 2.5. Exploitation Phase (Sufficient Food)

When Q > 0.25 and Temp > 0.6, the snake moves to food only, and the corresponding equation is expressed as follows:

(10) $$\begin{array}{c} X_{i}\left(t+1\right)=X_{food}\pm c_{3}\times Temp\times rand\times (X_{food}-X_{i}(t)) \end{array}$$

where $c_{3}$ is constant and equals 2, $X_{food}$ refers to the position of food, ± operator is determined by a random number r ∈ [0,1]; if r < 0.5, the negative sign is used, and if r ≥ 0.5, the positive sign is used.

If Q > 0.25 and Temp < 0.6, the snake will be in the fight mode or mating mode.

(1) Fight Mode

The fight behavior of snakes is mathematically demonstrated in the following equations:

(11) $$X_{i,m}(t+1)=X_{i,m}(t)\pm c_{3}\times F_{m}\times rand\times (Q\times X_{best,f}-X_{i,m}(t))$$

(12) $$X_{i,f}(t+1)=X_{i,f}(t)\pm c_{3}\times F_{f}\times rand\times (Q\times X_{best,m}-X_{i,f}(t))$$

where $X_{i,m}$, $X_{i,f}$ denote the positions of male and female individuals, respectively. ± operator is determined by a random number r ∈ [0,1]; if r < 0.5, the negative sign is used, and if r ≥ 0.5, the positive sign is used. $F_{m}$ and $F_{f}$ are the fighting abilities of male and female snakes, given as follows:

(13) $$F_{m}=exp\left(\frac{-f_{best,f}}{f_{i}}\right)$$

(14) $$F_{f}=exp\left(\frac{-f_{best,m}}{f_{i}}\right)$$

where $f_{i}$ is the fitness of the ith individual; $f_{best,f}$ and $f_{best,m}$ refer to the fitness of the best individual in female and male populations.

(2) Mating Mode

Regarding the snake mating behavior, the snake positions are updated as follows:

(15) $$X_{i,m}(t+1)=X_{i,m}(t)+c_{3}\times M_{m}\times rand\times (Q\times X_{i,f}(t)-X_{i,m}(t))$$

(16) $$X_{i,f}(t+1)=X_{i,f}(t)+c_{3}\times M_{f}\times rand\times (Q\times X_{i,m}(t)-X_{i,f}(t))$$

$M_{m}$ and $M_{f}$ are the mating abilities of male and female snakes, computed as follows:

(17) $$M_{m}=exp\left(\frac{-f_{i,f}}{f_{i,m}}\right)$$

(18) $$M_{f}=exp\left(\frac{-f_{i,m}}{f_{i,f}}\right)$$

The worst male and female members of the population are replaced if snake eggs hatch, according to Equations (19) and (20):

(19) $$X_{worst,m}=X_{\min}+rand\times (X_{\max}-X_{\min})$$

(20) $$X_{worst,f}=X_{\min}+rand\times (X_{\max}-X_{\min})$$

$X_{worst,m}\;\&\;X_{worst,f}$ refer to the worst male and female snakes, respectively.

Control parameters are summarized as follows in Table 1.

## 3. Modified Snake Optimizer (MSO)

Although the Snake Optimizer (SO) has demonstrated promising optimization performance across various applications, it still faces many challenges related to the quality of the initial population, convergence speed, and escaping the risk of local optima [15]. To overcome these limitations, this study proposes a Modified Snake Optimizer (MSO) that systematically enhances the original SO through the integration of multiple complementary strategies.

### 3.1. Dual Mapping Strategy Based on Latin Hypercube Sampling and Logistic Mapping

The quality of the initial population significantly influences the global optimal solution. In the SO algorithm, using random numbers to initialize the population frequently results in an uneven spread of solutions, which diminishes the ability to locate global optima [16].

To address this problem, a dual mapping strategy based on Latin hypercube sampling and logistic mapping is introduced to improve the distribution of the population.

Latin hypercube sampling (LHS) is a sampling method for multidimensional parameter spaces [17]. Its core principle involves uniformly partitioning the variable distribution range into multiple non-overlapping subintervals, then performing independent, equal-probability sampling within each subinterval. This ensures that sample points uniformly cover the entire distribution space. Unlike traditional random sampling methods, LHS employs a stratified strategy to better distribute sample points across the entire interval. Therefore, a mapping method based on LHS is introduced to initialize the position.

The equation for calculating the initial population position based on LHS is as follows:

(21) $$X_{i}=ub+lhs\times (lb-ub)$$

where lhs is the random sequence generated by Latin hypercube sampling, and ub and lb denote the upper and lower bounds of the search space, respectively.

To further improve the quality of the population, we also consider using chaotic mapping to generate another initial population.

Chaotic motion is characterized by randomness, ergodicity, and a high sensitivity to initial conditions [18]. Logistic mapping, a common nonlinear dynamical equation that models complex chaotic behavior, is used for population initialization because of its simplicity and strong randomness. It is expressed as follows:

(22) $$X_{i+1}=a*X_{i}*(1-X_{i})$$

where a is the control parameter in the interval  (0,4]. The logistic map enters the chaotic regime when a > 3.57, and a = 4 corresponds to a fully chaotic state. This study sets a to 4 for enhancing population diversity.

Using Latin hypercube sampling and the logistic mapping, individuals are generated separately. These individuals are then combined, and the top N individuals based on fitness are selected as the initial population.

Unlike existing SO variants that only use chaotic initialization or metaheuristics with LHS only, the proposed hybrid initialization strategy integrates the advantages of both methods. It ensures the initial population covers the entire search space uniformly while enhancing diversity, which directly addresses the initialization limitation of the original SO.

### 3.2. Opposition-Based Learning Mechanism with Scaling Factors

During the exploration phase of the Snake Optimizer, individuals update their positions by randomly selecting points within the search space. This method frequently results in a population with limited diversity and quality, which can hinder the ability to locate the global optimization.

Incorporating opposite-based learning strategy into swarm intelligence algorithms has been shown to improve their performance [19]. To overcome the limitations in exploration ability, this paper proposes an opposition-based learning mechanism with scaling factors. This mechanism broadens the search coverage and augments population diversity, thereby significantly enhancing the algorithm’s global search capability.

Firstly, the central positions of the male and female populations are determined, then two random scaling factors are introduced to generate mirror solutions. The fitness values of the mirror solutions and the original individuals are calculated, and the individual with the better (lower) fitness is chosen as a new position. The corresponding equations are as follows:

(23) $$X_{mirror,m}={center}_{m}+alpha\times ({center}_{m}-X_{m})$$

(24) $$X_{mirror,f}=center_{f}+beta\times (center_{f}-X_{f})$$

where $center_{m}$ and $center_{f}$ are the center positions of the current female and male populations, alpha and beta are scaling factors, calculated as follows:

(25) alpha=0.7+0.6×rand

(26) beta=0.7+0.6×rand

By incorporating scaling factors into the opposition-based learning mechanism, the proposed strategy effectively expands the search range and improves population diversity during the exploration phase.

### 3.3. Exploitation Strategy for the Integrated Soft-Rime Search Strategy from RIME

In conditions characterized by abundant food availability (Q) and excessively elevated temperatures (Temp), snakes only consume existing food—mathematically represented in the algorithm as convergence toward the current optimal solution. This process relies heavily on guidance from the current optimal position, lacking sufficient random perturbations or global jump mechanisms. If the optimal solution is unstable, the algorithm risks becoming confined to local optima.

To enhance the algorithm’s exploitation performance and avoid getting stuck in local optima, the soft-rime search strategy from RIME optimization is incorporated into the exploitation phase of MSO to generate higher-quality new candidate solutions.

RIME is a nature-inspired intelligent optimization algorithm derived from the physical process of rime-ice formation [20]. By simulating the growth processes of soft-rime and hard-rime, this algorithm searches for optimal solutions within the solution space, demonstrating excellent global search capabilities. The soft-rime search strategy factor RF is expressed as:

(27) $$RF=(r-0.5)\times 2\times \cos((\frac{\pi \cdot t}{10\cdot T}))\times (1-round(\frac{t\times W}{T})/W)$$

where r is a random number between 0 and 1, t is the current iteration number, T is the maximum iteration number, and W is a constant equal to 5.

This strategy is integrated into the hot environment exploitation phase of the MSO algorithm, with the relevant formulas as follows:

(28) $$X_{i,m}=X_{food}\pm C_{3}\times Temp\times rand\times RF$$

(29) $$X_{i,f}=X_{food}\pm C_{3}\times Temp\times rand\times RF$$

Equations (28) and (29) are mathematically consistent with identical parameter meanings, corresponding to the position update formulas for male and female snakes, respectively. ± operator is determined by a random number r ∈ [0,1]; if r < 0.5, the negative sign is used, and if r ≥ 0.5, the positive sign is used. Subsequently, with a probability of 50%, the algorithm determines whether to accept the new candidate solutions that integrate the soft-rime growth strategy factor RF or the solutions generated by the original SO algorithm. This approach maintains the exploitation capability of the original SO algorithm while mitigating the risk of convergence to local optima.

The adaptive nature of the soft-rime search strategy factor RF enables MSO to dynamically adjust exploitation step sizes as the search progresses. Unlike fixed local search strategies used in SO and many hybrid algorithms, this mechanism complements the original SO framework and provides greater flexibility in balancing convergence speed and solution quality.

In summary, the proposed Modified Snake Optimizer (MSO) algorithm effectively addresses the inherent drawbacks of the original Snake Optimizer (SO) and its existing variants—including insufficient initial population diversity, insufficient global search capability, and inefficient local exploitation—by introducing three tailored improvement strategies and systematically integrating them into the SO framework. Unlike the simple superposition of isolated techniques adopted in most existing hybrid metaheuristic approaches, MSO’s integration is designed to complement SO’s inherent snake-inspired search mechanism and target its core limitations synergistically.

### 3.4. Algorithm Flow

The implementation procedure of the MSO algorithm comprises the following steps:

Step 1: Establish a mathematical model based on the problem to be solved and define the objective function.

Step 2: Set the snake population size and maximum iteration number. Initialize snake positions using a dual mapping strategy based on Latin hypercube sampling and logistic mapping.

Step 3: Divide the population into male and female groups. Define the fitness function, calculate corresponding fitness values, and identify the current best male/female snakes and the global optimum.

Step 4: Define the food quantity and environmental temperature.

Step 5: Determine whether to enter the exploration or exploitation phase based on food quantity Q. If Q < 0.25, the snake finds food and updates the position using the opposite-based learning mechanism with scaling factors.

Step 6: When Q > 0.25 and Temp > 0.6, snakes consume existing food. Generate new positions using either the original SO algorithm or new equations incorporating the soft-rime search strategy from RIME, selected with a 50% probability.

Step 7: When Q > 0.25 and Temp < 0.6, use a pattern random number to choose between fight mode or mating mode.

Step 8: Evaluate the newly generated individual and refine the historical optimum accordingly.

Step 9: The algorithm evaluates the termination criterion based on the current and maximum number of iterations. If this limit is not reached, the process proceeds; otherwise, terminate the iteration and output the optimal position.

**Figure 1:**
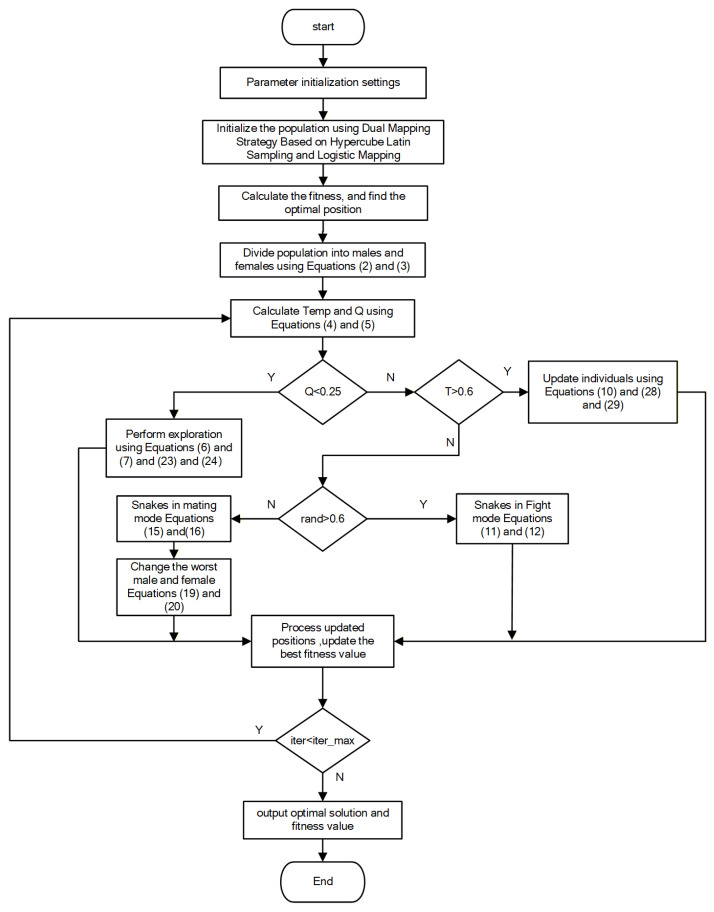
Algorithm flow of MSO.

The workflow of the MSO algorithm is illustrated in Figure 1 below.

## 4. Algorithm Comparison and Result Analysis

To comprehensively validate the effectiveness of the MSO in solving mathematical and engineering application problems, the proposed MSO method was applied to 30 benchmark functions from CEC 2017 [21], three engineering application problems, and two three-dimensional UAV path planning scenarios. Experimental outcomes were benchmarked against SO [10] alongside eight other state-of-the-art algorithms: BWO [7], DBO [8], CPO [9], COA [22], HHO [23], MFO [24], AOA [25], and WOA [26]. The simulation test environment configuration is as follows: operating system: Windows 11, 64-bit version; processor: 13th Gen Intel Core (TM) i5-13500H, base frequency 2.60 GHz; system equipped with 32 GB RAM; simulation software used: MATLAB R2024b. For all algorithms, parameter settings followed the recommendations provided in their original publications. The detailed parameter configurations of the comparison algorithms are summarized in Table 2.

### 4.1. Results on CEC 2017

This section presents a comprehensive evaluation to verify the performance and efficiency of the MSO algorithm. We use 30 functions from CEC 2017 for comparative experiments. These benchmark functions impose considerable challenges on optimization algorithms, categorized into four types: unimodal, multi-peak, hybrid, and composite functions. Specific details can be found in the relevant literature [21].

To ensure a fair and statistically meaningful comparison, all algorithms were executed with a population size of 30 and a maximum of 500 iterations. Simulations were carried out in three distinct dimensions—30, 50, and 100—to assess the efficacy and scalability of the MSO algorithm in resolving optimization problems of different dimensions. Considering the stochastic characteristics of metaheuristic algorithms, each algorithm underwent 30 independent runs to provide a statistically significant performance evaluation. The average value (AVG) and standard deviation (STD) were used as the primary performance indicators, as commonly adopted for assessing algorithm performance [27].

**Figure 2:**
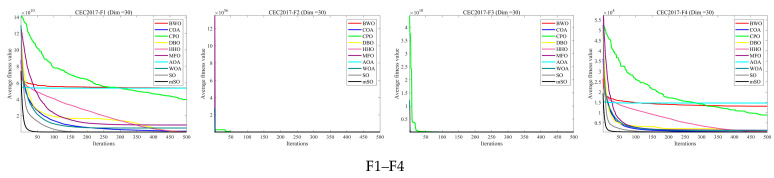

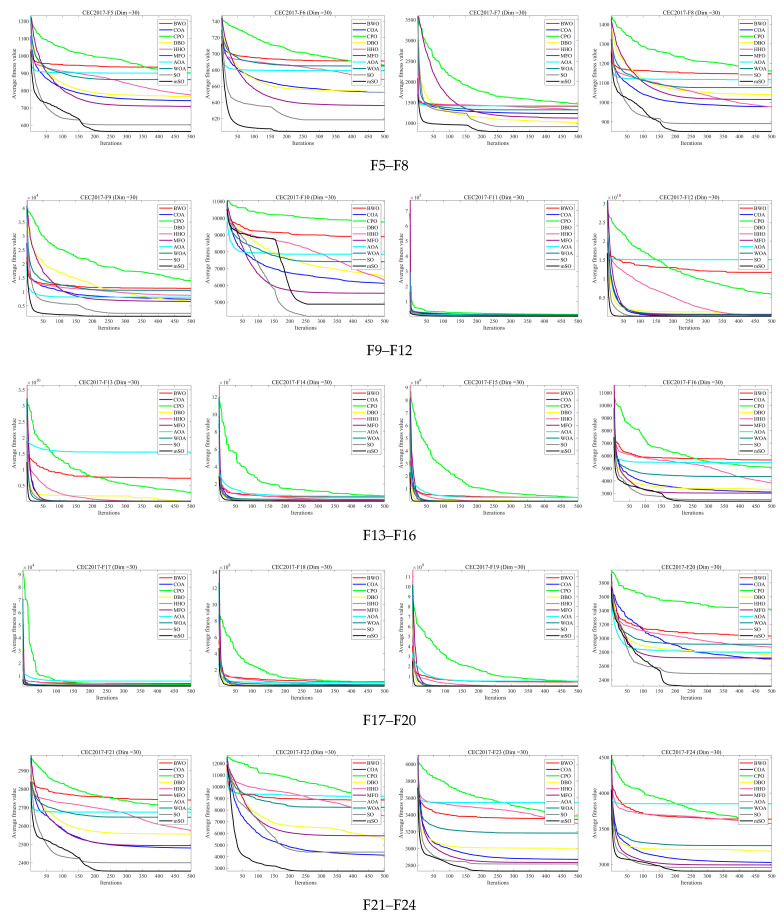

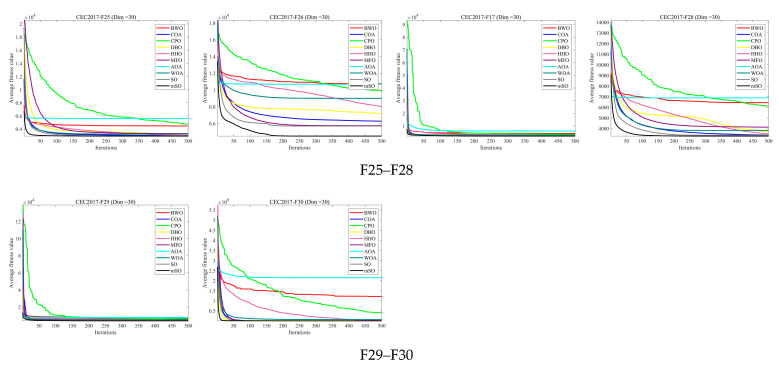
Convergence curve of 30 functions for all algorithms using CEC2017 and Dim = 30. Note: “mSO” in the figures refers to the proposed Modified Snake Optimizer (MSO) algorithm.

**Figure 3:**
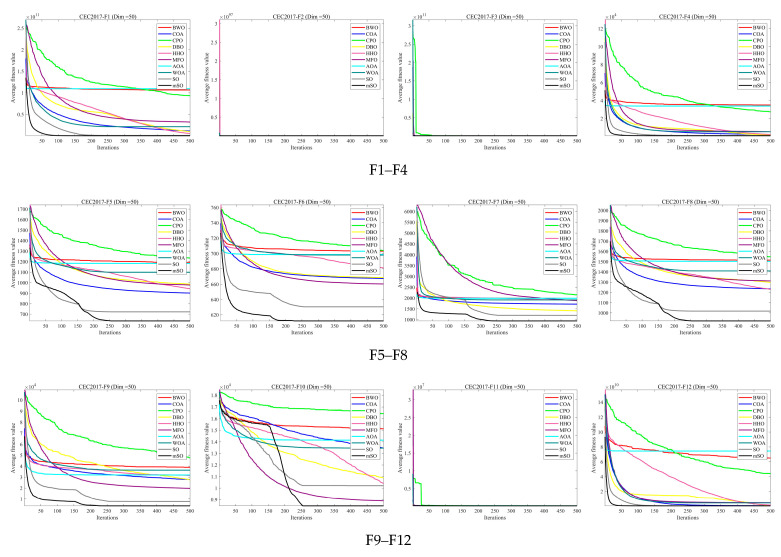

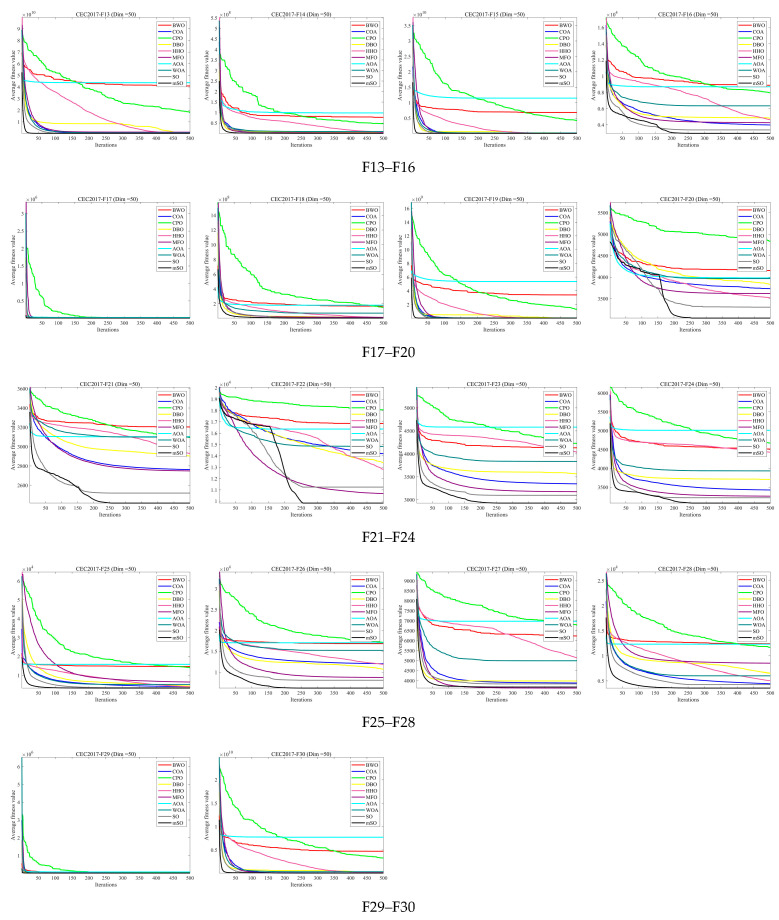
Convergence curve of 30 functions for all algorithms using CEC2017 and Dim = 50.

**Figure 4:**
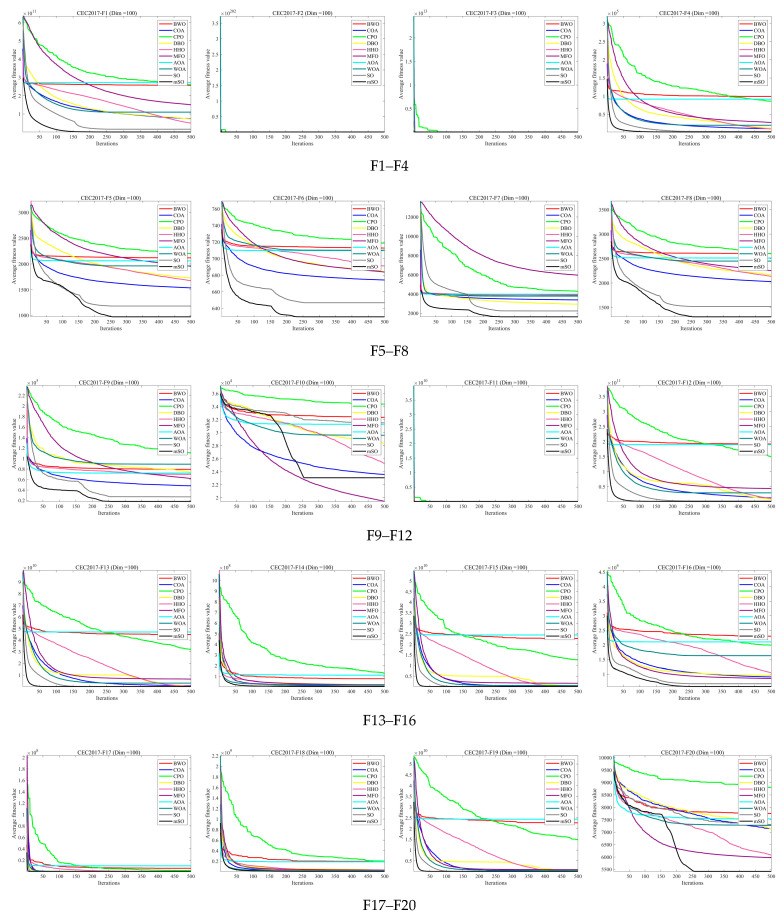

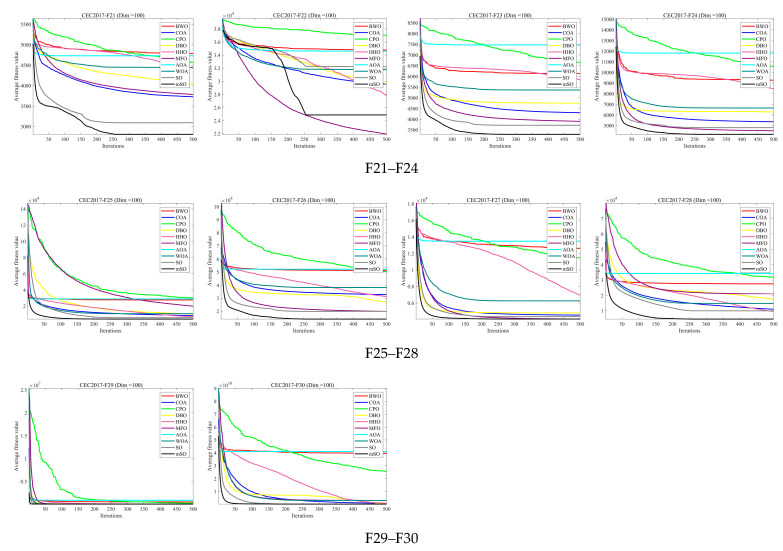
Convergence curve of 30 functions for all algorithms using CEC2017 and Dim = 100.

Figure 2, Figure 3 and Figure 4 illustrate the convergence curves of 30 benchmark functions at dimensions 30, 50, and 100. Each curve represents the average convergence behavior over 30 independent runs. In most benchmark functions, the MSO algorithm converges faster than the other compared algorithms and attains superior solutions, highlighting its strong capability to identify global optima. Overall, the convergence curves demonstrate that the MSO algorithm exhibits notable superiority in solving optimization problems.

**Figure 5:**
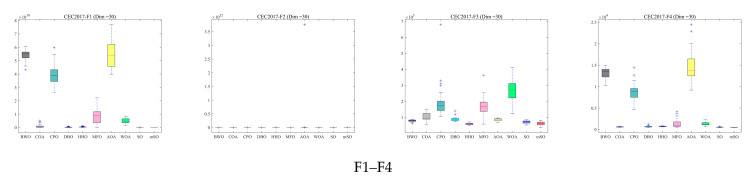

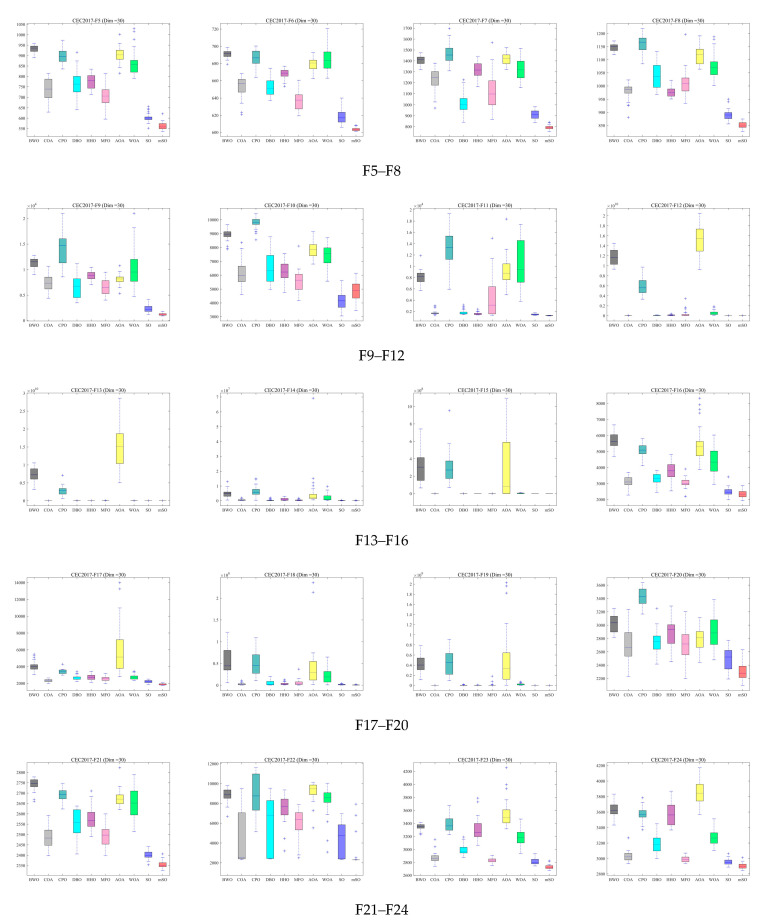

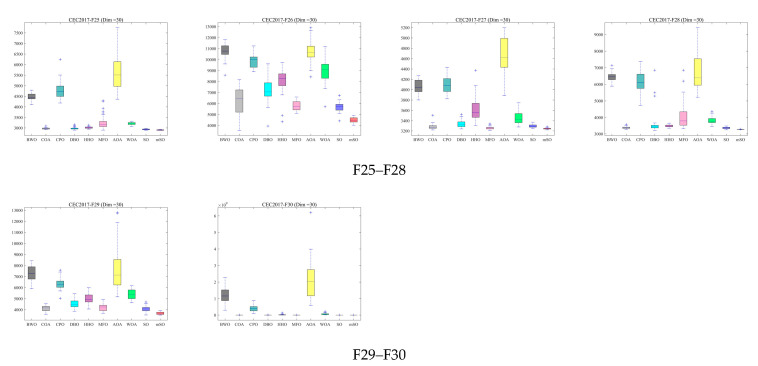
Box plot of 30 functions for all algorithms using CEC2017 and Dim = 30.

**Figure 6:**
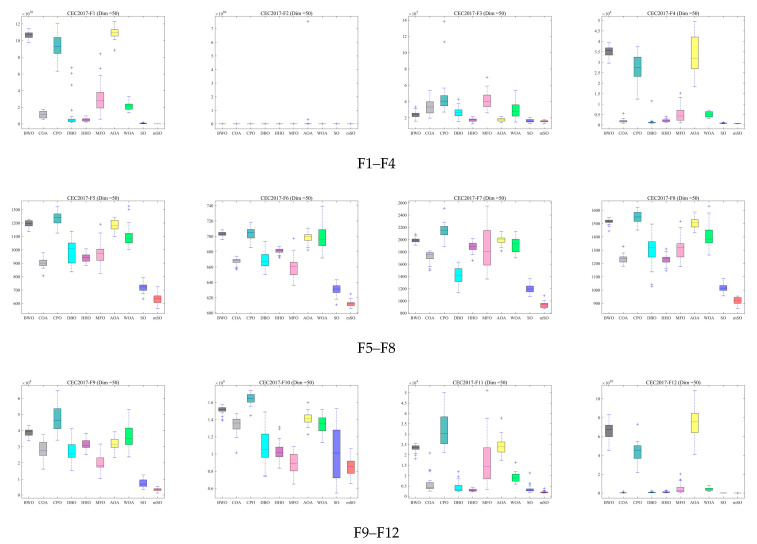

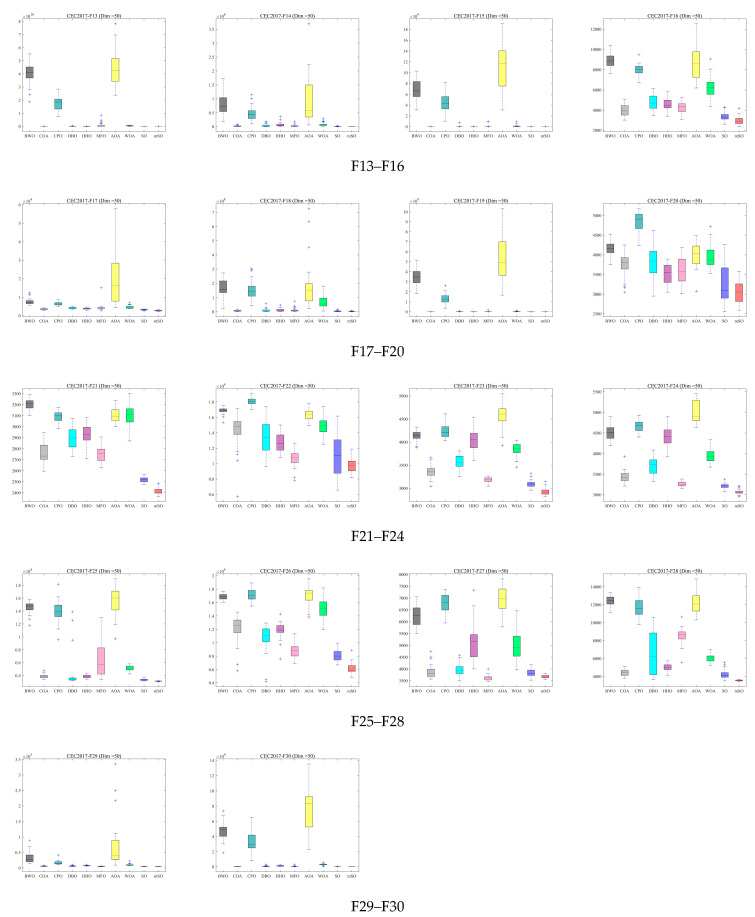
Box plot of 30 functions for all algorithms using CEC2017 and Dim = 50.

**Figure 7:**
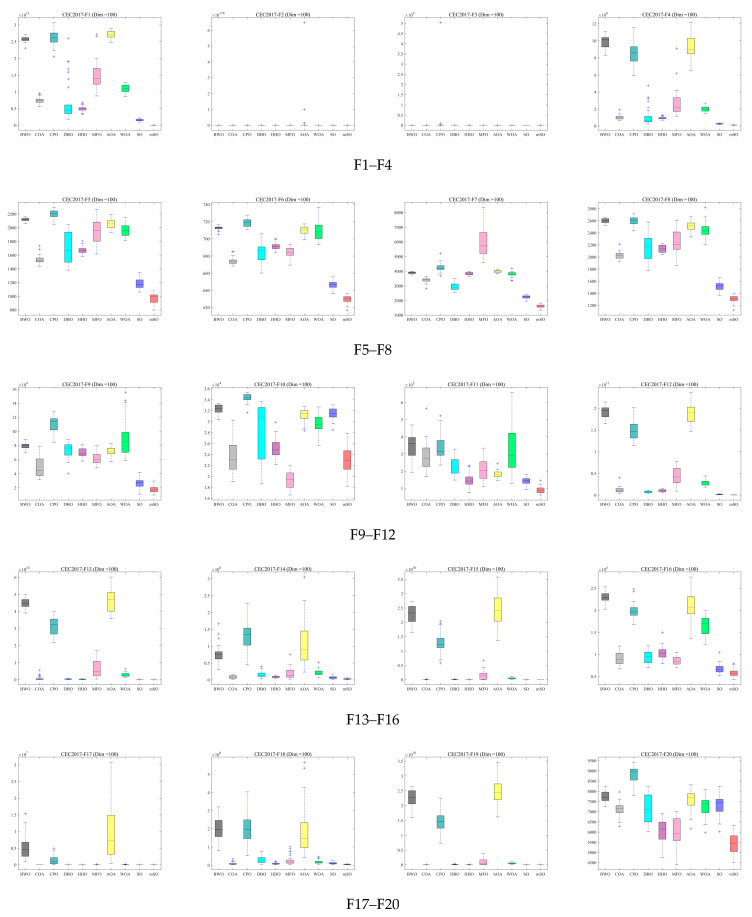

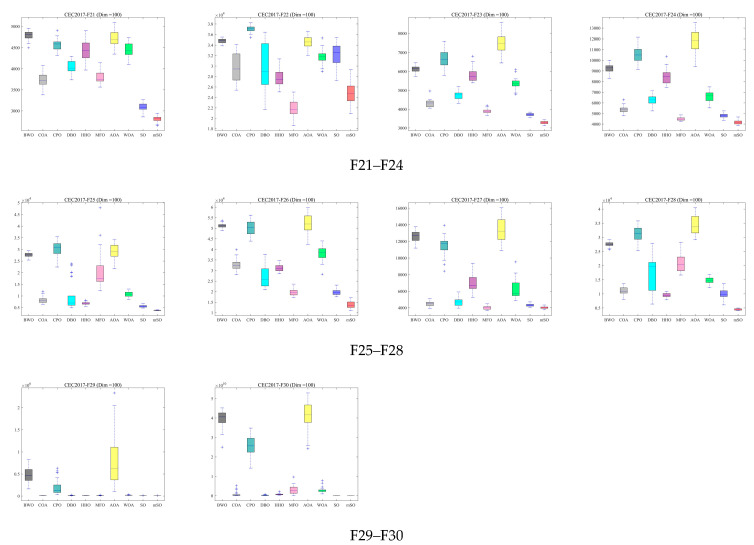
Box plot of 30 functions for all algorithms using CEC2017 and Dim = 100.

To further assess robustness and stability, box plots of the optimization results across three dimensions are presented in Figure 5, Figure 6 and Figure 7. These box plots summarize the distribution characteristics of the solutions obtained over 30 independent runs for each benchmark function. For the majority of benchmark functions, the MSO algorithm achieves superior median performance, narrower interquartile ranges, and fewer outliers. These results show that MSO attains higher solution accuracy and exhibits significantly greater robustness and stability compared with alternative algorithms, including the original SO algorithm.

Table 3 reports the detailed numerical comparison results of all algorithms across the 30 benchmark functions under three different dimensions, evaluated using the AVG and STD. These numerical experiments clearly demonstrate that the MSO outperforms the competing algorithms.

Specifically, from the perspective of the average performance, when the dimension is set to 30, the MSO algorithm attained the top performance on 24 benchmark functions and secured second place on the remaining six functions. The HHO and SO algorithms obtained the best performance on one and five benchmark functions, respectively. At a dimension of 50, the MSO algorithm ranked first on 28 functions, while in the remaining two, it ranked second, with the SO algorithm achieving the optimal mean performance. When the dimension increased to 100, the MSO algorithm secured the top mean values on 28 functions, while the MFO and SO algorithms each ranked first on one function. The results indicate that the MSO algorithm shows excellent optimization performance and strong stability across problems of varying dimensionality.

Regarding solution stability, as measured by the standard deviation, when the dimension is set to 30, the MSO algorithm ranked first in 23 benchmark functions, second in five, third in one, and fifth in one, while the standard SO algorithm ranked first in two functions. At a dimension of 50, the MSO algorithm ranked first in 17 functions, whereas the BWO and SO algorithms achieved the best performance in seven and five functions respectively. When the dimensionality increased to 100, the MSO algorithm achieved the lowest standard deviation across 19 test functions, while the SO algorithm achieved it for two functions. The BWO algorithm also demonstrated competitive performance, obtaining the lowest standard deviation for eight functions, and the CPO algorithm ranked first in one benchmark function. These results show that the MSO exhibits remarkable stability and consistency in addressing optimization problems across different dimensions. In summary, when addressing optimization problems across varying dimensions, the MSO algorithm consistently exhibits outstanding stability and reliability.

Table 4 presents the Friedman test results and the corresponding rankings of the MSO compared with nine other algorithms on the CEC2017 benchmark set. The results show that the MSO algorithm consistently achieved superior (lower) ranking values across all three dimensions (30, 50, and 100), ultimately attaining the top average rank. These outcomes highlight the effectiveness of the enhancement strategies introduced in the study. Furthermore, it is evident that the advantages of the MSO algorithm become increasingly pronounced as the problem dimensionality grows, demonstrating its superior optimization capability and scalability.

To statistically validate the performance differences between MSO and the competing algorithms, including SO, the Wilcoxon rank-sum test with a 0.05 significance level was conducted. The detailed results are presented in Table 5, Table 6 and Table 7. A *p*-value lower than 0.05 implies rejection of the null hypothesis, demonstrating a statistically significant difference in performance.

As indicated in Table 5, Table 6 and Table 7, the MSO algorithm exhibits statistically significant performance differences with respect to the original SO and the other eight algorithms across most test functions. Together with the Friedman test results, these results underscore the distinctive characteristics of MSO in addressing optimization problems.

To evaluate the individual contributions of the three enhancement strategies incorporated in MSO, ablation experiments were conducted. Specifically, the effectiveness and necessity of each strategy were examined by comparing MSO with the original SO and three algorithmic variants, each with one strategy removed.

1. MSO_A: Dual mapping strategy is removed.
2. MSO_B: Opposition-based learning mechanism is removed.
3. MSO_C: Soft-rime search strategy is removed.

**Figure 8:**
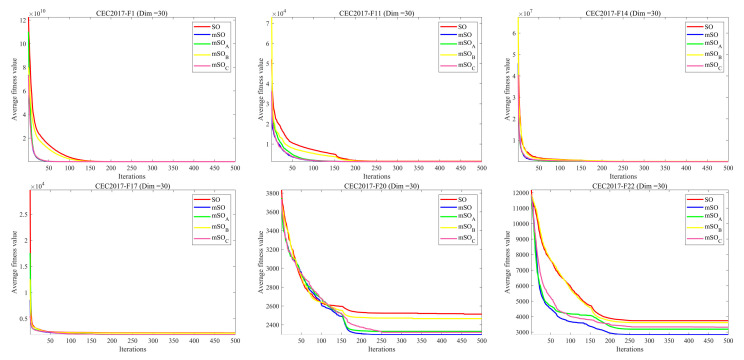
Convergence curves of the ablation experiment.

Six representative benchmark functions from the CEC2017 suite (F1, F11, F14, F17, F20, and F22) were selected to evaluate algorithmic performance. These functions represent diverse optimization scenarios with different levels of complexity, thereby enabling a comprehensive assessment of adaptability and robustness. The statistical results are summarized in Table 8, and the corresponding convergence behaviors are depicted in Figure 8.

For these benchmark functions, each variant achieves a certain level of improvement compared with the original SO algorithm. Notably, when all three strategies are jointly incorporated, MSO consistently attains lower mean values and standard deviations than both the individual variants and SO. This observation indicates that MSO delivers superior overall optimization performance, further confirming the synergistic effectiveness of the proposed multi-strategy fusion.

Overall, the results confirm that the MSO algorithm exhibits high efficiency and robustness. It effectively improves population diversity while maintaining superior global and local search capability and more rapid convergence. Moreover, the algorithm effectively avoids premature convergence to local optima and consistently delivers high-quality solutions across a wide range of optimization problems.

In summary, the MSO algorithm demonstrates a significant improvement in optimization performance compared with the original SO. When benchmarked against nine contemporary metaheuristic algorithms on the CEC 2017 test set, MSO consistently obtained superior results across various problems. The comprehensive statistical analyses, including convergence curves, box plots, Friedman rankings, and the Wilcoxon rank-sum test, confirm that MSO achieves superior accuracy and convergence efficiency while maintaining strong stability and robustness, with performance differences that are statistically significant. Furthermore, the ablation experiment demonstrates the effectiveness and synergistic benefits of each improvement strategy. These findings validate that the proposed modifications effectively address the inherent deficiencies of the standard SO framework, making MSO a highly competitive method for addressing complex, high-dimensional optimization challenges. Moreover, the outstanding performance on these theoretical benchmarks provides a solid foundation for extending MSO to real-world engineering design problems.

### 4.2. Real-World Engineering Design Problems

Real-world engineering design problems present greater challenges to algorithm performance, particularly in terms of stability and robustness. In this study, the proposed MSO algorithm is applied to solve three engineering design problems: pressure vessel design, tension/compression spring design, and hydrostatic thrust bearing design [28,29,30]. All algorithms employed a population size of 30 and 500 iterations, and each algorithm was independently executed 30 times to ensure a fair performance comparison. The definitions and constraints of these engineering problems are as follows:

#### 4.2.1. Pressure Vessel Design

Pressure vessel design can be formulated as a constrained engineering problem involving four variables: shell thickness ($x_{1}$), head thickness ($x_{2}$), inner radius ($x_{3}$), and vessel length ($x_{4}$). The objective is to minimize the total production cost of the pressure vessel.

The mathematical formulations and constraints of this problem are defined as follows:

(30) $$f\left(x\right)=0.6224x_{1}x_{3}x_{4}+1.7781x_{2}x_{3}^{2}+3.1661x_{1}^{2}x_{4}+19.84x_{1}^{2}x_{3}$$

Subject to:

(31) $$\begin{array}{l} g_{1}(x)=-x_{1}+0.0193x_{3}\leq 0\\ g_{2}(x)=-x_{2}+0.00954x_{3}\leq 0\\ g3(x)=-\pi {x_{3}}^{2}x_{4}-(4/3)\pi {x_{3}}^{3}+1296000\leq 0\\ g_{4}(x)=x_{4}-240\leq 0 \end{array}$$

As shown in Table 9, the MSO algorithm demonstrates exceptional overperformance by achieving the highest rank in the Friedman test. The worst-case result obtained by MSO was 6.37E+03, while its best-case value reached 6.04E+03. Furthermore, the MSO algorithm achieved the lowest average value of 6.28E+03 and the smallest standard deviation of 1.34E+02, indicating minimal variability, the highest stability and consistency. The DBO algorithm also exhibited competitive performance, but its relatively high standard deviation and average value prevented it from outperforming MSO. Similarly, the original Snake Optimizer produced competitive results but, with a standard deviation of 3.47E+02 (nearly three times that of MSO), ultimately ranked second, reflecting lower stability and consistency compared with the MSO algorithm.

#### 4.2.2. Tension/Compression Spring Design

The main objective of the problem is to minimize the overall weight of a tension or compression spring. The optimization problem involves four constraints and three design variables: wire diameter ($x_{1}$), average diameter of the spring coil ($x_{2}$), and total count of active coils ($x_{3}$). The mathematical formulation and corresponding constraints are presented as follows.

(32) $$f(x)=(x_{3}+2)x_{2}x_{1}^{2}$$

Subject to:

(33) $$\begin{array}{l} g_{1}(x)=1-\frac{x_{2}^{2}x_{3}}{71785x_{1}^{4}}\leq 0\\ g_{2}(x)=\frac{4{x_{2}}^{2}-x_{1}x_{2}}{12566(x_{2}{x_{1}}^{3}-x_{1}4)}+\frac{1}{5108{x_{1}}^{2}}-1\leq 0\\ g_{3}(x)=1-\frac{140.45x_{1}}{x_{2}^{2}x_{3}}\leq 0\\ g_{4}(x)=(x_{1}+x_{2})/1.5-1\leq 0 \end{array}$$

As shown in Table 10, the MSO algorithm once again demonstrates its superiority by achieving the best performance across most evaluation metrics—worst-case, average, median, and standard deviation—and securing the top position in the Friedman ranking. Among the compared algorithms, COA exhibited the most comparable performance, which nonetheless lags behind MSO in all the aforementioned metrics. Although the original algorithm identifies the optimal solution with a slight advantage, its standard deviation is nearly five times larger than that of MSO, indicating severely limited engineering reproducibility, a conclusion further supported by the Friedman test results. In contrast, the MSO algorithm consistently produces stable, high-quality solutions, making it more suitable for practical engineering applications.

Overall, the MSO algorithm significantly enhances stability, robustness and overall performance while maintaining a theoretical optimization ceiling comparable to the original SO algorithm. The statistical analysis confirms its superior comprehensive performance and suitability for real-world engineering applications demanding high robustness.

#### 4.2.3. Hydrostatic Thrust Bearing Design

The primary objective of this problem is to minimize power loss by optimizing four key parameters: oil viscosity ($x_{1}$), bearing radius ($x_{2}$), flow rate ($x_{4}$), and groove radius ($x_{4}$). The objective function and associated constraints are expressed as follows:

(34) $$f(x)=\frac{\frac{x_{3}p_{0}}{0.7}+E_{f}}{12}$$

Subject to:

(35) $$\begin{array}{l} g_{1}(x)=1000-p_{0}\leq 0\\ g_{2}(x)=w-101000\leq 0\\ g_{3}(x)=5000-\frac{w}{\pi \left({x_{2}}^{2}-{x_{4}}^{2}\right)}\leq 0\\ g_{4}(x)=50-p_{0}\leq 0\\ g_{5}(x)=0.001-\frac{0.0307}{386.4p_{0}}\left(\frac{x_{3}}{2\pi x_{2}h}\right)\leq 0\\ g_{6}(x)=x_{2}-x_{4}\leq 0\\ g_{7}(x)=h-0.001\leq 0 \end{array}$$

where

(36) $$\begin{array}{l} p_{0}=\frac{6x_{1}x_{3}}{\pi h^{3}}\ln\left(\frac{x_{2}}{x_{4}}\right)\\ w=\frac{\pi p_{0}}{2}\frac{x_{2}^{2}-x_{4}^{2}}{\ln\left(\frac{x_{2}}{x_{4}}\right)}\\ E_{f=}9336x_{3}^{*}0.0307^{*}0.5delT,delT=2(10^{p}-559.7)\\ p=\frac{\log_{10}\log_{10}\left(8.122*10^{6}x_{1}+0.8\right)+3.55}{10.04}\\ h={\left(\frac{2\pi^{*}750}{60}\right)}^{2}\frac{2\pi x_{1}}{E_{f}}\left(\frac{x_{2}^{4}}{4}-\frac{x_{4}^{4}}{4}\right) \end{array}$$

As shown in Table 11, the MSO algorithm exhibits outstanding performance and ranks first in the Friedman test. Its maximum, average, median, and standard deviation values all rank among the best, reflecting exceptional accuracy and reliability. The MSO algorithm’s standard deviation of 2.18E+02 indicates minimal variability and excellent consistency in results. The original SO algorithm also exhibits competitive performance in identifying optimal solutions, but it shows higher variability and lower consistency, as evidenced by its second-place ranking in the Friedman test. Despite the DBO algorithm achieving the optimal solution, its standard deviation of 2.12E+08 reveals poor stability and extremely low reproducibility, which limits its applicability to practical engineering problems. The remaining algorithms lag considerably behind MSO across all metrics, displaying significantly higher standard deviations and weaker performance stability. Overall, the remarkable results achieved by MSO in both accuracy and stability confirm its efficiency and reliability in solving engineering design problems.

Overall, the results obtained from the three engineering design problems clearly demonstrate the superior optimization performance of the proposed MSO algorithm. In each case, MSO consistently produced high-quality, stable, and reproducible solutions, outperforming the compared algorithms in terms of convergence speed, solution precision, and robustness. These findings confirm that MSO not only achieves effective global exploration and local exploitation but also maintains strong stability when addressing nonlinear, constrained engineering optimization problems. Consequently, the algorithm shows significant promise for solving practical engineering applications that require both accuracy and reliability. To further validate its effectiveness in handling complex spatial optimization tasks, the MSO algorithm is next applied to two three-dimensional UAV path planning scenarios with different objective functions and constraint conditions.

### 4.3. Three-Dimensional UAV Path Planning

UAV path planning is a well-established problem in the engineering optimization domain, where the complexity of three-dimensional environments poses significant challenges to algorithmic efficiency and robustness. To address three-dimensional UAV path planning, the study incorporates optimality criteria and cost function constraints tailored to UAV operational requirements. To more comprehensively validate the effectiveness of the MSO algorithm in handling this complex problem, two distinct 3D scenarios are examined, each characterized by different optimization objectives and constraints [31]. In these experiments, each algorithm employs a population size of 30 and 500 iterations, and is independently executed 30 times for a fair performance comparison.

#### 4.3.1. Three-Dimensional UAV Path Planning (Case 1)

For efficient UAV operation, the planned flight path should satisfy the optimality conditions defined by the specific mission requirements. For tasks such as surveying, surface inspection, and aerial photography, the main objective is to optimize the total path distance. The UAV’s trajectory is represented as a sequence of waypoints, and the cost $F_{1}$ associated with the path length is determined by calculating the Euclidean distance between consecutive waypoints, as defined below:

(37) $$F_{1}(X_{i})=\sum_{j=1}^{n-1}\vec{\left\|P_{ij}P_{i,j+1}\right\|}$$

where, $X_{i}$ is the ith flight trajectory, represented as a sequence of n waypoints, and $P_{ij}$ is the coordinate of the jth waypoint in the ith trajectory.

**Figure 9:**
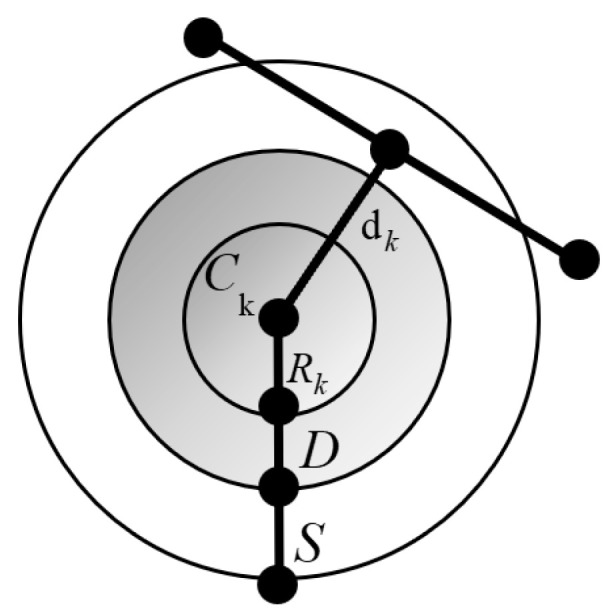
Determination of the threat cost.

Beyond minimizing path length, the flight trajectory must also guarantee operational safety by keeping the UAV at a sufficient distance from potential hazards, typically obstacles located within the mission environment. Let K represent the collection of all potential obstacles, each represented by a cylindrical model characterized by its center coordinates $C_{k}$ and radius $R_{k}$, as illustrated in Figure 9. For any given path segment $\vec{P_{ij}P_{i,j+1}}$, the magnitude of the threat cost depends on the distance $d_{k}$ between the UAV and the obstacle’s center point $C_{k}$. By incorporating the UAV’s body diameter D and an additional safety margin S surrounding the collision boundary, the cost function $F_{2}$ is defined as

(38) $$\left\{\begin{array}{l} F_{2}(X_{i})=\sum \begin{array}{l} n-1\\ j=1 \end{array}\sum \begin{array}{l} k\\ k=1 \end{array}T_{k}(\vec{P_{ij}P_{i,j+1}})\\ T_{k}(\vec{P_{ij}P_{i,j+1}})=\left\{\begin{array}{l} 0,ifd_{k}>S+D+R_{K}\\ (S+D+R_{K})-d_{k},ifD+R_{K}<d_{k}\leq S+D+R_{K}\\ \infty ,ifd_{k}\leq S+D+R_{K} \end{array}\right. \end{array}\right.$$

**Figure 10:**
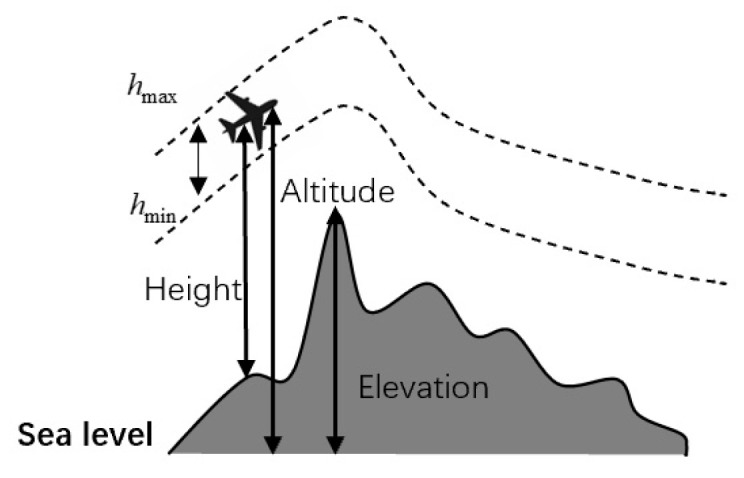
Altitude cost explanation.

The UAV’s flight altitude is typically constrained between predefined lower and upper bounds. In some practical applications, such as aerial surveying or search operations, visual data collection using cameras with specific resolutions and fields of view imposes supplementary constraints on the allowable altitude. $h_{min}$ and $h_{max}$ are defined as the upper and lower permissible altitude limits, respectively. The corresponding altitude cost of $P_{ij}$ is computed as follows:

(39) $$H_{ij}=\left\{\begin{array}{l} \left|h_{ij}-\frac{h_{max}+h_{min}}{2}\right|,\;ifh_{min}\leq h_{ij}\leq h_{max}\\ \infty ,otherwise \end{array}\right.$$

where $h_{ij}$ represents the flight altitude relative to the ground, as shown in Figure 10. This equation encourages the UAV to maintain an average height and imposes penalties when deviations from the allowable range. The total altitude cost $F_{3}$ is obtained by summing the altitude costs across all path points:

(40) $$F_{3}(X_{i})=\sum_{j=1}^{n}H_{ij}$$

**Figure 11:**
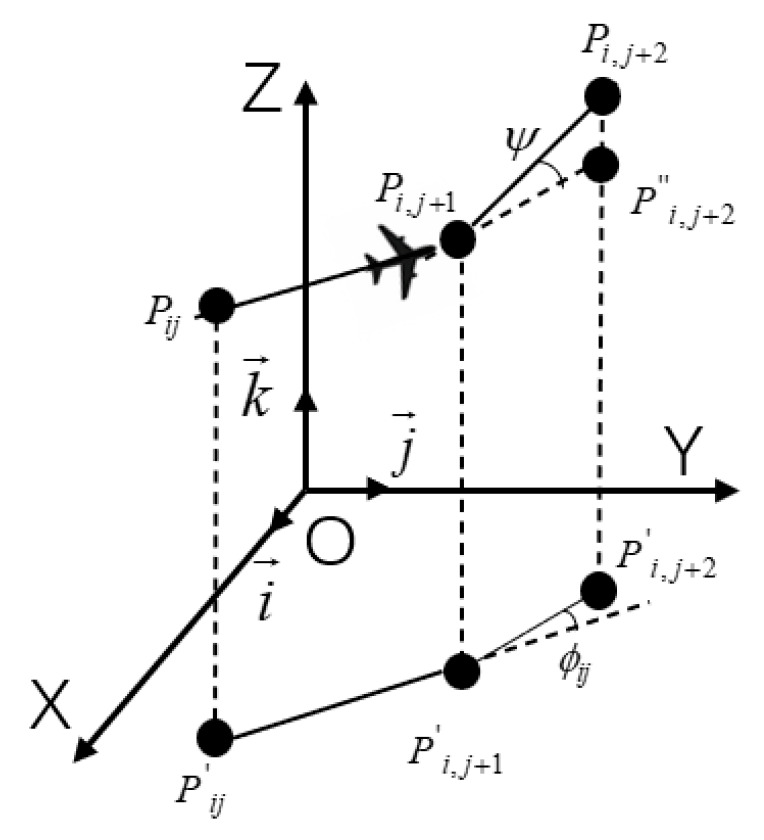
Turning and climbing angle.

Flight smoothness evaluates the UAV’s turning and climbing rates, which are critical for maintaining reliable and stable flight paths. As shown in Figure 11, the turn angle $\varphi_{ij}$ between two path segments, $\vec{{P^{\prime }}_{ij}{P^{\prime }}_{i,j+1}}$ and $\vec{{P^{\prime }}_{ij+1}{P^{\prime }}_{i,j+2}}$, is projected onto the horizontal plane (Oxy).

$\vec{k}$ is defined as the unit vector oriented along the z-axis; accordingly, the projection of the vector onto the horizontal plane can be expressed as

(41) $$\vec{{P^{\prime }}_{ij}{P^{\prime }}_{i,j+1}}=\vec{k}\times (\vec{P_{ij}P_{i,j+1}},\vec{k})$$

Thus, the turn angle $\varphi_{ij}$ is computed as

(42) $$\varphi_{ij}=arctan\left(\frac{\left\|\vec{{P^{\prime }}_{ij}{P^{\prime }}_{i,j+1}}\times \vec{{P^{\prime }}_{i,j+1}{P^{\prime }}_{i,j+2}}\right\|}{\vec{{P^{\prime }}_{ij}{P^{\prime }}_{i,j+1}}\cdot \vec{{P^{\prime }}_{i,j+1}{P^{\prime }}_{i,j+2}}}\right)$$

The climb angle $\psi_{ij}$ denotes the angle between a path segment and its horizontal projection, expressed as

(43) $$\psi_{ij}=arctan\left(\frac{z_{i,j+1}-z_{ij}}{\left\|\vec{{P^{\prime }}_{ij}{P^{\prime }}_{i,j+1}}\right\|}\right)$$

Thus, the smoothness cost is calculated as

(44) $$F_{4}(X_{i})=a_{1}\sum_{j=1}^{n-2}\varphi_{ij}+a_{2}\sum_{j=1}^{n-1}|\psi_{ij}-\psi_{i,j-1}|$$

$a_{1}$ and $a_{2}$ denote the weights for turn and climb angles, both equal to 1. Based on the four factors, the overall cost of the UAV is formulated as

(45) $$F(X_{i})=\sum_{k=1}^{4}b_{k}F_{k}(X_{i})$$

where $b_{k}$ denotes the weighting coefficient, and the decision variables, and $X_{i}$ consists of $P_{ij}$ defined within the unmanned aerial vehicle airspace.

In this study, we set the weights $b_{1}$, $b_{2}$, $b_{3}$ and $b_{4}$ to 5, 1, 10, and 1. The simulation defines the initial position of the UAV as Start = (100, 100, 150) and the destination as End = (800, 800, 150).

**Figure 12:**
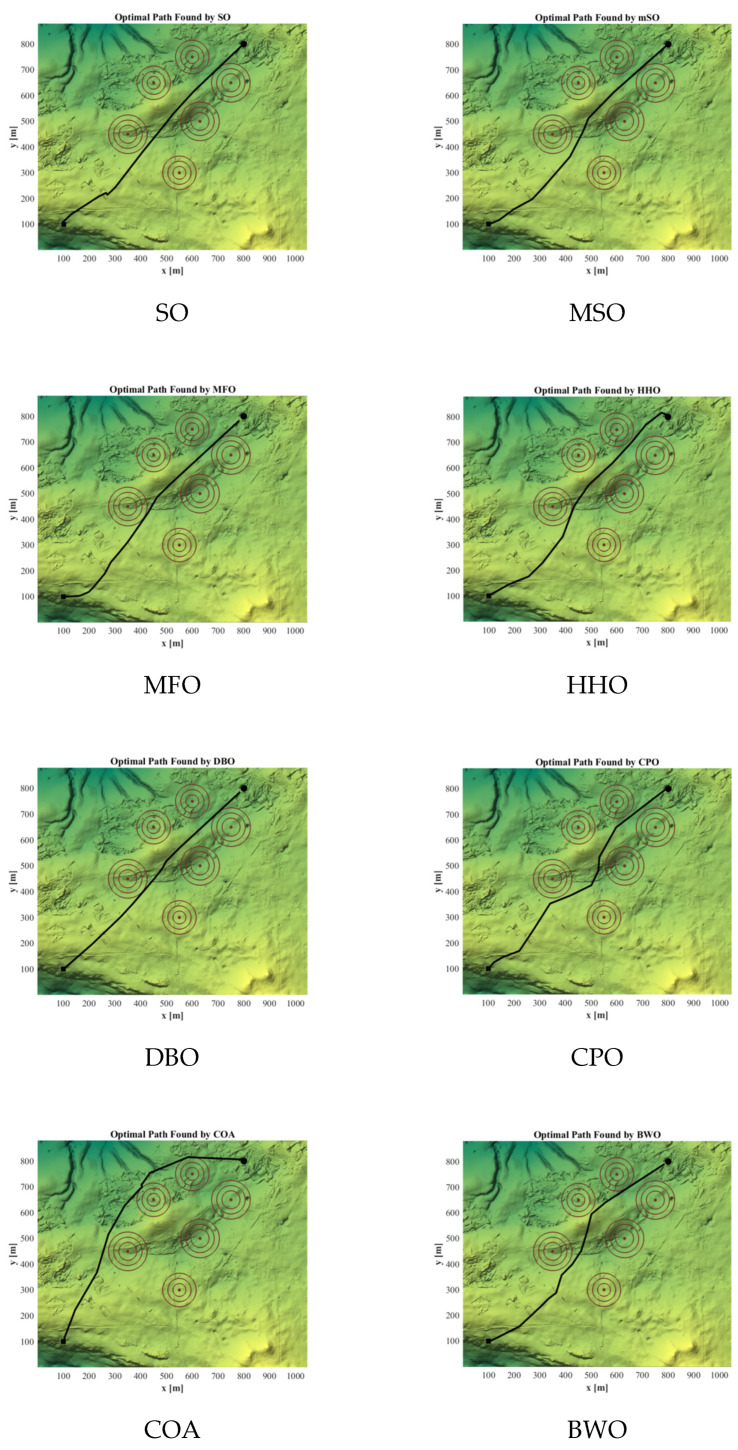

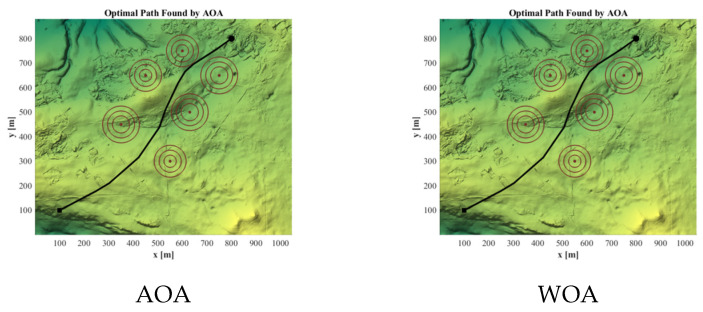
Visualization of three-dimensional UAV path planning results (case 1).

Figure 12 visualizes the optimal trajectories obtained by all algorithms. The MSO-derived trajectory maintains an adequate clearance from obstacles and exhibits improved smoothness, resulting in a shorter and safer flight path compared to the alternatives.

As shown in Table 12, the MSO algorithm exhibits superior overall performance and ranks first in the Friedman test. Although its best minimum value (5.09E+03) is not the best among all comparative algorithms, MSO demonstrates the highest consistency across evaluation metrics by achieving the optimal maximum value, mean, median, and standard deviation simultaneously. These results highlight its strong stability and minimal performance variability over 30 independent runs.

Overall, the MSO algorithm clearly outperforms the compared algorithms in both stability and comprehensive optimization capability, making it a more reliable and robust choice for the UAV path planning problem.

#### 4.3.2. Three-Dimensional UAV Path Planning (Case 2)

Using cubic spline interpolation, a smooth curve comprising g points is generated. The objective function corresponding to this problem is formulated as follows:

(46) $$F=a_{1}\times F_{pc}+a_{2}\times F_{hc}+a_{3}\times F_{sc}$$

where F represents the total cost, $F_{pc}$ denotes the cost corresponding to path length,$F_{hc}$ indicates the cost related to the standard deviation of flight height, and $F_{sc}$ signifies the smoothness cost.

Optimizing the path length is essential for minimizing flight time and operational costs. The mathematical model is expressed as follows:

(47) $$F_{pc}=\sum_{m=1}^{g-1}{\left\|(x_{m+1},y_{m+1},z_{m+1})-(x_{m},y_{m},z_{m})\right\|}_{2}$$

where $\left(x_{m},y_{m},z_{m}\right)$ represents the three-dimensional position coordinate corresponding to the mth point of the UAV.

The UAV’s flight altitude plays a crucial role in determining flight stability and operational safety; therefore, it must be carefully considered in the total cost. The altitude-related cost model is defined mathematically as

(48) $$F_{hc}=\sqrt{\sum_{m=1}^{g}{\left(z_{m}-\frac{1}{n}\sum_{k=1}^{g}z_{m}\right)}^{2}}$$

Finally, the effect of UAV turning maneuvers on flight smoothness must be taken into account, and the corresponding mathematical formula is given as follows:

(49) $$F_{sc}=\sum_{m=1}^{g-2}\left(arccos\left(\frac{\varphi_{m+1}\times \varphi_{m}}{\left|\varphi_{m+1}|\times |\varphi_{m}\right|}\right)\right)$$

where $\varphi_{m}$ denotes $\left(x_{m+1}-x_{m},y_{m+1}-y_{m},z_{m+1}-z_{m}\right)$.

The obstacle set is defined by the following equation. During flight, the UAV must avoid these obstacles, as illustrated below:

(50) $$z=sin(y+1)+sin(x)+cos(x^{2}+y^{2})+2\times cos(y)+sin(x^{2}+y^{2})$$

For the total cost function F, we set the weights $a_{1}$, $a_{2}$, and $a_{3}$ to 0.4, 0.4, and 0.2, respectively, with corresponding start pzzoint S = $\left(0,0,20\right)$ and end point E = $\left(200,200,20\right)$.

**Figure 13:**
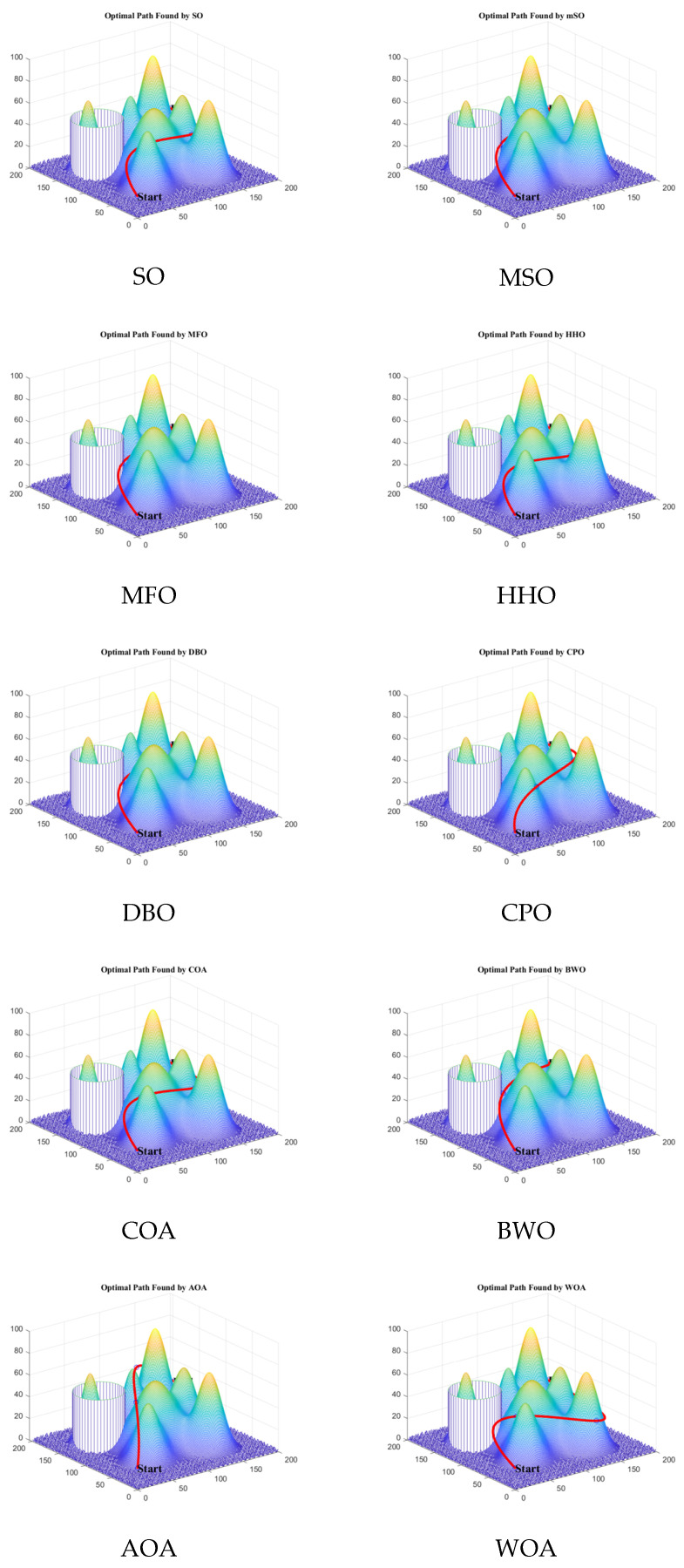
Visualization of 3D UAV path planning results (case 2).

Figure 13 illustrates the optimal paths generated by ten different algorithms, demonstrating that the proposed MSO algorithm identifies a shorter and smoother path under the given conditions.

Table 13 presents the statistical analysis results of three-dimensional UAV path planning (case 2). It is evident that the MSO algorithm achieved the best overall performance across all evaluation metrics—maximum, minimum, mean, median, and standard deviation—demonstrating exceptional stability and minimal variability. Moreover, the MSO algorithm ranks first in the Friedman test, further confirming its strong competitiveness, stability, and robustness in solving complex engineering optimization problems.

## 5. Conclusions

To address the limitations of the original Snake Optimizer (SO)—including insufficient population diversity, weak exploration capability, and a tendency to become trapped in local optima—a Modified Snake Optimizer (MSO) is proposed. First, a dual mapping strategy based on Latin hypercube sampling and logistic mapping is introduced to generate a more uniformly distributed initial population, thereby accelerating convergence. During the exploration phase, an opposition-based learning mechanism with scaling factors is employed to expand the search range and enhance global optimization performance. In the exploitation phase, the soft-rime search strategy from RIME is integrated to strengthen local search ability and effectively prevent premature convergence.

Comprehensive experiments were conducted using 30 benchmark functions from CEC2017 at dimensions of 30, 50, and 100, comparing MSO with nine advanced metaheuristic algorithms, including the original SO. The results consistently illustrate that MSO outperforms other comparative algorithms in terms of convergence speed, overall robustness, and optimization accuracy. To validate the capability to address engineering application problems, the MSO algorithm was applied to three engineering design problems and two UAV path planning scenarios. Across all cases, MSO produced stable, high-quality, and reproducible solutions, highlighting its engineering robustness, practical reliability, and broad applicability.

Overall, the proposed MSO algorithm effectively extends the biomimetic principles of the original SO by improving both search efficiency and solution stability. Owing to its simple structure, outstanding performance, and strong adaptability, MSO represents a practical, biomimetically inspired optimization framework suitable for both theoretical studies and engineering applications. Future research will explore hybridization with other bio-inspired mechanisms and adaptive parameter control to further enhance adaptability and performance in dynamic and multi-objective optimization environments. In addition, in-depth theoretical analysis will be pursued to complement the experimental findings presented in this study.

## Figures and Tables

**Table 1:** Control parameters specifications.

Control Parameters	Value	Role
$c_{1}$	0.5	calculate food quantity
$c_{2}$	0.05	control exploration intensity
$c_{3}$	2	control exploitation intensity

**Table 2:** Algorithm parameter settings.

Algorithm	Parameterization
BWO	$B_{f}=B_{0}\cdot (1-\frac{t}{T}),\;C_{1}=2\cdot r_{4}\cdot (1-\frac{t}{T}),\;\beta =1.5,\;C_{2}=2\cdot W_{f}\cdot N,\;W_{f}=0.1-0.05\cdot \frac{t}{T}$
DBO	$a=\pm 1,\;b\in (0,1),\;k\in (0,0.02],\;R=1-\frac{t}{T},\;C_{1}\sim N(0,1),\;C_{2}\in (0,1)$
CPO	$T=2,\;a=0.2,\;T_{f}=0.8$
COA	I=1or2, r∈(0,1)
HHO	$E=2\cdot E_{0}\cdot (1-\frac{t}{T}),\;E_{0}\in [-1,1]$
MFO	t∈[−1,1], b=1
AOA	μ=0.499, a=5
WOA	$A=2\cdot a\cdot r_{1}-a,\;C=2\cdot r_{2},\;a=2-2\cdot \frac{t}{T},\;b=1$
SO	$c_{1}=0.5,\;c_{2}=0.05,\;c_{3}=2$
MSO	$c_{1}=0.5,\;c_{2}=0.05,\;c_{3}=2$

**Table 3:** The comparison results of all algorithms over 30 functions on 3 dimension values.

No.	Dim	Metric	BWO	COA	CPO	DBO	HHO	MFO	AOA	WOA	SO	MSO
F1	30	STD	3.79E+09	1.34E+09	8.27E+09	2.17E+08	2.94E+08	5.32E+09	1.01E+10	1.95E+09	1.96E+07	**4.57E+04**
		AVG	5.38E+10	9.99E+08	3.98E+10	2.57E+08	4.39E+08	8.66E+09	5.37E+10	4.94E+09	1.53E+07	**1.80E+05**
	50	STD	4.42E+09	4.21E+09	1.41E+10	1.70E+10	2.06E+09	1.87E+10	7.48E+09	5.04E+09	3.12E+08	**4.28E+05**
		AVG	1.07E+11	1.12E+10	9.34E+10	9.73E+09	5.25E+09	3.22E+10	1.10E+11	2.13E+10	6.65E+08	**1.67E+06**
	100	STD	7.43E+09	9.51E+09	2.13E+10	6.21E+10	7.66E+09	4.26E+10	1.08E+10	1.21E+10	3.33E+09	**1.27E+08**
		AVG	2.57E+11	7.39E+10	2.60E+11	7.17E+10	4.99E+10	1.50E+11	2.71E+11	1.10E+11	1.55E+10	**1.91E+08**
F2	30	STD	9.90E+45	8.61E+27	1.09E+44	6.95E+32	1.31E+35	4.27E+40	6.85E+51	9.55E+37	3.00E+27	**2.30E+19**
		AVG	3.11E+45	1.58E+27	2.12E+43	1.53E+32	3.55E+34	7.84E+39	1.26E+51	3.02E+37	9.20E+26	**6.86E+18**
	50	STD	1.74E+81	1.42E+59	1.91E+81	9.45E+71	1.88E+69	2.31E+74	1.38E+86	3.11E+81	6.76E+58	**3.56E+40**
		AVG	4.00E+80	2.60E+58	3.58E+80	2.09E+71	3.45E+68	4.36E+73	2.64E+85	5.72E+80	1.24E+58	**7.23E+39**
	100	STD	6.55E+04	5.02E+149	6.55E+04	6.55E+04	6.55E+04	6.55E+04	6.55E+04	**6.55E+04**	2.11E+134	6.26E+116
		AVG	5.34E+172	9.17E+148	1.37E+174	2.76E+153	2.03E+154	9.39E+163	2.59E+177	9.84E+171	7.24E+133	**1.14E+116**
F3	30	STD	6.94E+03	2.39E+04	1.08E+05	1.30E+04	5.71E+03	5.66E+04	7.79E+03	7.07E+04	8.63E+03	**1.21E+04**
		AVG	7.87E+04	1.03E+05	1.96E+05	8.83E+04	**5.88E+04**	1.72E+05	8.52E+04	2.67E+05	7.13E+04	6.03E+04
	50	STD	3.92E+04	8.51E+04	2.33E+05	6.33E+04	2.26E+04	9.93E+04	1.91E+04	9.53E+04	1.92E+04	**1.31E+04**
		AVG	2.40E+05	3.35E+05	4.60E+05	2.64E+05	1.72E+05	4.15E+05	1.76E+05	2.97E+05	1.62E+05	**1.56E+05**
	100	STD	5.36E+04	1.17E+05	9.19E+08	2.43E+05	1.32E+05	1.46E+05	4.85E+04	1.43E+05	3.61E+04	**2.71E+04**
		AVG	3.81E+05	7.05E+05	1.73E+08	6.08E+05	3.77E+05	9.94E+05	3.77E+05	8.88E+05	3.81E+05	**3.55E+05**
F4	30	STD	1.15E+03	5.79E+01	2.08E+03	1.51E+02	8.16E+01	9.66E+02	3.51E+03	3.87E+02	3.96E+01	**2.18E+01**
		AVG	1.32E+04	6.06E+02	8.82E+03	6.76E+02	7.20E+02	1.39E+03	1.47E+04	1.32E+03	5.51E+02	**5.02E+02**
	50	STD	2.62E+03	9.30E+02	6.05E+03	1.91E+03	6.36E+02	3.85E+03	8.38E+03	1.36E+03	1.37E+02	**4.61E+01**
		AVG	3.50E+04	1.98E+03	2.75E+04	1.51E+03	2.07E+03	5.25E+03	3.39E+04	5.13E+03	8.66E+02	**6.17E+02**
	100	STD	7.33E+03	2.64E+03	1.29E+04	1.09E+04	1.47E+03	1.61E+04	1.37E+04	3.51E+03	5.33E+02	**8.22E+01**
		AVG	9.94E+04	1.01E+04	8.65E+04	1.12E+04	9.50E+03	2.74E+04	9.18E+04	1.99E+04	2.59E+03	**1.10E+03**
F5	30	STD	**1.82E+01**	5.35E+01	3.50E+01	5.98E+01	3.53E+01	4.83E+01	3.74E+01	6.17E+01	2.12E+01	1.89E+01
		AVG	9.33E+02	7.42E+02	8.99E+02	7.65E+02	7.75E+02	7.09E+02	9.01E+02	8.63E+02	6.02E+02	**5.63E+02**
	50	STD	**2.51E+01**	3.32E+01	4.69E+01	8.84E+01	3.40E+01	7.61E+01	3.51E+01	7.58E+01	3.26E+01	3.92E+01
		AVG	1.19E+03	9.01E+02	1.23E+03	9.86E+02	9.41E+02	9.78E+02	1.18E+03	1.10E+03	7.22E+02	**6.35E+02**
	100	STD	**2.43E+01**	5.96E+01	6.64E+01	2.29E+02	4.69E+01	1.74E+02	6.82E+01	9.15E+01	6.85E+01	7.39E+01
		AVG	2.12E+03	1.54E+03	2.20E+03	1.72E+03	1.68E+03	1.95E+03	2.06E+03	1.96E+03	1.18E+03	**9.70E+02**
F6	30	STD	4.59E+00	1.23E+01	9.44E+00	1.00E+01	5.79E+00	1.12E+01	7.40E+00	1.35E+01	7.73E+00	**2.22E+00**
		AVG	6.91E+02	6.53E+02	6.86E+02	6.53E+02	6.68E+02	6.36E+02	6.79E+02	6.85E+02	6.18E+02	**6.04E+02**
	50	STD	**3.30E+00**	4.33E+00	7.32E+00	1.13E+01	3.76E+00	1.46E+01	6.89E+00	1.34E+01	7.00E+00	3.90E+00
		AVG	7.03E+02	6.67E+02	7.04E+02	6.69E+02	6.81E+02	6.60E+02	6.99E+02	6.98E+02	6.31E+02	**6.12E+02**
	100	STD	2.43E+00	**4.01E+00**	4.80E+00	1.10E+01	4.05E+00	6.85E+00	4.66E+00	1.25E+01	4.75E+00	4.66E+00
		AVG	7.13E+02	6.74E+02	7.19E+02	6.84E+02	6.91E+02	6.84E+02	7.10E+02	7.10E+02	6.47E+02	**6.30E+02**
F7	30	STD	3.80E+01	1.01E+02	8.09E+01	8.89E+01	7.34E+01	1.61E+02	5.34E+01	8.87E+01	4.20E+01	**1.98E+01**
		AVG	1.41E+03	1.23E+03	1.46E+03	1.01E+03	1.32E+03	1.12E+03	1.42E+03	1.32E+03	9.13E+02	**7.92E+02**
	50	STD	**4.01E+01**	9.01E+01	1.12E+02	1.32E+02	8.19E+01	3.57E+02	6.28E+01	1.16E+02	7.57E+01	4.91E+01
		AVG	1.99E+03	1.72E+03	2.15E+03	1.41E+03	1.88E+03	1.89E+03	1.99E+03	1.92E+03	1.20E+03	**9.37E+02**
	100	STD	**6.47E+01**	1.70E+02	2.95E+02	2.59E+02	8.96E+01	9.97E+02	8.21E+01	1.72E+02	1.18E+02	1.16E+02
		AVG	3.89E+03	3.40E+03	4.29E+03	2.95E+03	3.83E+03	5.96E+03	3.98E+03	3.79E+03	2.24E+03	**1.62E+03**
F8	30	STD	1.49E+01	2.98E+01	3.54E+01	5.16E+01	1.84E+01	4.97E+01	3.40E+01	4.77E+01	2.04E+01	**1.29E+01**
		AVG	1.15E+03	9.78E+02	1.16E+03	1.04E+03	9.77E+02	1.02E+03	1.12E+03	1.08E+03	8.91E+02	**8.49E+02**
	50	STD	**2.06E+01**	3.09E+01	4.57E+01	1.11E+02	3.32E+01	8.47E+01	3.57E+01	8.43E+01	2.87E+01	2.58E+01
		AVG	1.51E+03	1.23E+03	1.55E+03	1.30E+03	1.23E+03	1.31E+03	1.50E+03	1.41E+03	1.02E+03	**9.20E+02**
	100	STD	**3.50E+01**	5.63E+01	7.38E+01	2.25E+02	5.99E+01	1.87E+02	8.08E+01	1.25E+02	6.78E+01	5.97E+01
		AVG	2.60E+03	2.03E+03	2.60E+03	2.17E+03	2.14E+03	2.25E+03	2.51E+03	2.45E+03	1.52E+03	**1.30E+03**
F9	30	STD	1.00E+03	1.61E+03	3.08E+03	2.01E+03	9.10E+02	1.61E+03	1.05E+03	4.08E+03	7.49E+02	**2.38E+02**
		AVG	1.13E+04	7.40E+03	1.41E+04	6.61E+03	8.82E+03	6.58E+03	8.14E+03	1.06E+04	2.30E+03	**1.23E+03**
	50	STD	2.27E+03	5.23E+03	8.21E+03	6.10E+03	3.20E+03	5.12E+03	3.42E+03	7.01E+03	2.51E+03	**8.38E+02**
		AVG	3.90E+04	2.79E+04	4.77E+04	2.75E+04	3.15E+04	1.98E+04	3.20E+04	3.64E+04	7.59E+03	**3.52E+03**
	100	STD	**4.36E+03**	1.34E+04	1.20E+04	1.05E+04	5.92E+03	8.34E+03	6.20E+03	2.58E+04	6.52E+03	4.38E+03
		AVG	7.94E+04	4.80E+04	1.11E+05	7.37E+04	6.97E+04	6.19E+04	7.23E+04	8.92E+04	2.68E+04	**1.79E+04**
F10	30	STD	**4.06E+02**	8.85E+02	4.08E+02	1.14E+03	6.41E+02	8.24E+02	6.31E+02	8.65E+02	6.84E+02	6.65E+02
		AVG	8.90E+03	6.13E+03	9.75E+03	6.54E+03	6.27E+03	5.53E+03	7.85E+03	7.41E+03	**4.15E+03**	4.88E+03
	50	STD	4.42E+02	9.63E+02	6.46E+02	1.87E+03	1.10E+03	1.17E+03	7.15E+02	8.72E+02	3.13E+03	**1.01E+03**
		AVG	1.51E+04	1.34E+04	1.64E+04	1.09E+04	1.04E+04	8.88E+03	1.41E+04	1.35E+04	1.02E+04	**8.44E+03**
	100	STD	7.91E+02	2.95E+03	**7.65E+02**	5.03E+03	1.87E+03	1.59E+03	1.11E+03	1.63E+03	1.06E+03	2.49E+03
		AVG	3.23E+04	2.36E+04	3.43E+04	2.83E+04	2.52E+04	1.94E+04	3.13E+04	2.96E+04	3.15E+04	**2.31E+04**
F11	30	STD	1.20E+03	3.99E+02	3.18E+03	4.57E+02	2.36E+02	3.50E+03	2.65E+03	4.13E+03	1.26E+02	**2.96E+01**
		AVG	8.13E+03	1.75E+03	1.34E+04	1.82E+03	1.60E+03	4.59E+03	9.34E+03	1.04E+04	1.45E+03	**1.24E+03**
	50	STD	1.68E+03	3.93E+03	8.00E+03	2.44E+03	6.16E+02	1.18E+04	4.22E+03	2.20E+03	1.79E+03	**4.70E+02**
		AVG	2.32E+04	5.91E+03	3.22E+04	4.41E+03	3.00E+03	1.72E+04	2.44E+04	9.20E+03	3.49E+03	**1.97E+03**
	100	STD	7.22E+04	8.14E+04	8.12E+04	5.16E+04	3.89E+04	6.04E+04	2.15E+04	1.45E+05	**1.96E+04**	2.05E+04
		AVG	3.52E+05	2.91E+05	3.40E+05	2.28E+05	1.49E+05	2.10E+05	1.81E+05	3.30E+05	1.43E+05	**8.85E+04**
F12	30	STD	1.59E+09	9.08E+06	1.69E+09	5.51E+07	8.63E+07	7.14E+08	3.02E+09	4.46E+08	2.67E+06	3.30E+06
		AVG	1.17E+10	1.46E+07	5.89E+09	4.98E+07	1.17E+08	3.39E+08	1.51E+10	5.34E+08	**3.52E+06**	4.31E+06
	50	STD	9.49E+09	3.43E+08	1.04E+10	4.72E+08	6.89E+08	4.95E+09	1.60E+10	1.79E+09	3.25E+07	**1.25E+07**
		AVG	6.51E+10	2.56E+08	4.37E+10	7.58E+08	1.09E+09	4.44E+09	7.44E+10	4.13E+09	5.38E+07	**2.54E+07**
	100	STD	1.29E+10	7.07E+09	2.07E+10	2.32E+09	2.67E+09	1.86E+10	2.35E+10	6.31E+09	5.84E+08	**8.40E+07**
		AVG	1.92E+11	1.23E+10	1.50E+11	7.40E+09	1.03E+10	4.40E+10	1.90E+11	2.93E+10	1.74E+09	**3.15E+08**
F13	30	STD	2.01E+09	1.35E+05	1.33E+09	8.05E+06	4.98E+05	2.45E+07	5.83E+09	9.18E+06	**2.09E+04**	3.32E+04
		AVG	7.26E+09	1.83E+05	2.79E+09	4.31E+06	1.09E+06	1.06E+07	1.55E+10	1.14E+07	**3.78E+04**	7.61E+04
	50	STD	8.10E+09	1.28E+07	5.53E+09	8.61E+07	4.46E+07	1.92E+09	1.35E+10	2.65E+08	2.92E+05	**4.66E+04**
		AVG	4.09E+10	5.52E+06	1.81E+10	5.89E+07	4.48E+07	1.02E+09	4.37E+10	4.57E+08	3.01E+05	**1.07E+05**
	100	STD	2.69E+09	1.24E+09	5.06E+09	2.10E+08	1.51E+08	4.98E+09	6.64E+09	1.16E+09	8.28E+06	**6.21E+04**
		AVG	4.48E+10	7.57E+08	3.16E+10	3.42E+08	2.64E+08	6.53E+09	4.70E+10	2.99E+09	7.40E+06	**1.75E+05**
F14	30	STD	2.77E+06	5.48E+05	3.37E+06	4.95E+05	8.73E+05	3.98E+05	1.25E+07	2.52E+06	9.19E+04	**5.20E+04**
		AVG	4.86E+06	4.91E+05	6.32E+06	3.49E+05	1.00E+06	4.69E+05	5.78E+06	2.24E+06	7.72E+04	**3.60E+04**
	50	STD	3.46E+07	1.96E+06	2.56E+07	5.08E+06	7.50E+06	3.84E+06	8.33E+07	7.62E+06	7.39E+05	**1.64E+05**
		AVG	7.75E+07	1.69E+06	4.69E+07	4.04E+06	6.99E+06	2.90E+06	9.85E+07	8.86E+06	7.65E+05	**2.59E+05**
	100	STD	2.80E+07	4.65E+06	4.32E+07	1.04E+07	2.65E+06	1.63E+07	7.01E+07	9.25E+06	3.78E+06	**1.44E+06**
		AVG	7.77E+07	8.82E+06	1.30E+08	1.77E+07	9.32E+06	1.90E+07	1.12E+08	2.09E+07	7.56E+06	**3.00E+06**
F15	30	STD	1.74E+08	2.27E+04	1.79E+08	6.52E+04	5.00E+04	4.00E+04	3.60E+08	4.35E+06	9.76E+03	**8.68E+03**
		AVG	3.05E+08	2.73E+04	2.98E+08	7.96E+04	1.19E+05	4.70E+04	3.05E+08	4.34E+06	**1.36E+04**	1.93E+04
	50	STD	1.77E+09	1.31E+05	1.81E+09	1.39E+08	2.75E+06	2.30E+08	4.39E+09	1.90E+08	4.08E+04	**2.89E+04**
		AVG	6.85E+09	1.20E+05	4.27E+09	2.86E+07	2.32E+06	6.54E+07	1.15E+10	1.03E+08	4.22E+04	**3.94E+04**
	100	STD	3.07E+09	4.60E+07	3.56E+09	5.49E+07	9.78E+06	1.57E+09	5.90E+09	2.67E+08	2.12E+05	**1.65E+04**
		AVG	2.28E+10	2.32E+07	1.27E+10	4.54E+07	1.80E+07	1.58E+09	2.44E+10	5.00E+08	2.90E+05	**6.61E+04**
F16	30	STD	4.61E+02	3.75E+02	3.77E+02	3.36E+02	5.89E+02	3.33E+02	1.15E+03	7.54E+02	2.64E+02	**2.40E+02**
		AVG	5.66E+03	3.11E+03	5.09E+03	3.28E+03	3.82E+03	3.02E+03	5.48E+03	4.35E+03	2.50E+03	**2.37E+03**
	50	STD	6.47E+02	6.00E+02	6.11E+02	7.69E+02	6.24E+02	5.59E+02	1.57E+03	1.01E+03	4.39E+02	**3.99E+02**
		AVG	8.88E+03	3.98E+03	7.96E+03	4.80E+03	4.63E+03	4.26E+03	8.70E+03	6.35E+03	3.36E+03	**2.93E+03**
	100	STD	1.30E+03	1.36E+03	1.87E+03	1.36E+03	1.37E+03	9.03E+02	3.14E+03	2.18E+03	1.11E+03	**8.71E+02**
		AVG	2.30E+04	9.06E+03	1.99E+04	9.44E+03	1.05E+04	8.51E+03	2.10E+04	1.63E+04	6.68E+03	**5.71E+03**
F17	30	STD	5.88E+02	1.85E+02	2.64E+02	2.84E+02	3.48E+02	2.92E+02	2.87E+03	3.11E+02	1.72E+02	**1.02E+02**
		AVG	4.03E+03	2.33E+03	3.39E+03	2.68E+03	2.70E+03	2.55E+03	5.94E+03	2.72E+03	2.23E+03	**1.89E+03**
	50	STD	1.70E+03	3.77E+02	8.58E+02	4.78E+02	4.17E+02	2.04E+03	1.44E+04	7.37E+02	**2.41E+02**	2.88E+02
		AVG	7.64E+03	3.59E+03	6.55E+03	4.25E+03	3.81E+03	4.53E+03	2.06E+04	4.71E+03	3.23E+03	**2.81E+03**
	100	STD	3.76E+06	9.97E+02	1.37E+06	1.41E+03	1.22E+03	3.84E+04	8.78E+06	4.52E+04	**5.06E+02**	6.93E+02
		AVG	5.52E+06	6.90E+03	1.42E+06	9.12E+03	8.00E+03	1.73E+04	9.99E+06	3.68E+04	5.86E+03	**4.73E+03**
F18	30	STD	3.09E+07	2.49E+06	2.68E+07	6.44E+06	3.11E+06	7.52E+06	5.37E+07	1.74E+07	1.06E+06	**3.45E+05**
		AVG	5.63E+07	2.44E+06	5.04E+07	5.39E+06	3.46E+06	6.00E+06	4.44E+07	2.17E+07	1.18E+06	**3.65E+05**
	50	STD	5.73E+07	5.19E+06	6.86E+07	1.17E+07	1.06E+07	1.54E+07	1.62E+08	4.77E+07	4.05E+06	**1.45E+06**
		AVG	1.67E+08	7.63E+06	1.50E+08	1.04E+07	1.20E+07	1.33E+07	1.80E+08	7.13E+07	4.99E+06	**3.03E+06**
	100	STD	6.05E+07	7.41E+06	8.12E+07	1.58E+07	5.18E+06	2.70E+07	1.35E+08	1.05E+07	5.80E+06	**1.51E+06**
		AVG	1.99E+08	9.67E+06	2.03E+08	2.78E+07	9.13E+06	2.90E+07	1.90E+08	1.95E+07	1.13E+07	**4.28E+06**
F19	30	STD	1.84E+08	4.36E+04	2.35E+08	3.90E+06	1.52E+06	3.73E+07	5.68E+08	1.83E+07	2.91E+04	**1.57E+04**
		AVG	4.26E+08	3.24E+04	4.39E+08	2.21E+06	1.66E+06	1.24E+07	5.15E+08	2.03E+07	2.55E+04	**2.12E+04**
	50	STD	8.01E+08	2.63E+05	5.12E+08	1.36E+07	1.85E+06	3.31E+07	2.19E+09	2.02E+07	**5.03E+04**	6.84E+04
		AVG	3.41E+09	3.27E+05	1.31E+09	7.26E+06	2.42E+06	9.91E+06	5.35E+09	1.73E+07	**5.88E+04**	7.89E+04
	100	STD	2.77E+09	1.16E+07	3.48E+09	8.35E+07	2.12E+07	1.06E+09	4.38E+09	2.38E+08	2.42E+06	**1.41E+06**
		AVG	2.27E+10	1.52E+07	1.47E+10	9.02E+07	4.22E+07	8.20E+08	2.44E+10	5.17E+08	2.45E+06	**1.86E+06**
F20	30	STD	1.28E+02	2.36E+02	1.30E+02	1.83E+02	2.04E+02	2.18E+02	1.79E+02	2.20E+02	1.63E+02	**1.26E+02**
		AVG	3.03E+03	2.70E+03	3.42E+03	2.75E+03	2.87E+03	2.72E+03	2.80E+03	2.91E+03	2.49E+03	**2.31E+03**
	50	STD	**1.81E+02**	2.97E+02	2.38E+02	3.90E+02	2.51E+02	3.28E+02	2.87E+02	3.03E+02	5.39E+02	2.76E+02
		AVG	4.15E+03	3.73E+03	4.84E+03	3.83E+03	3.52E+03	3.62E+03	3.98E+03	3.97E+03	3.30E+03	**3.04E+03**
	100	STD	**2.75E+02**	3.58E+02	3.81E+02	7.24E+02	5.51E+02	7.28E+02	4.85E+02	5.07E+02	5.03E+02	4.97E+02
		AVG	7.75E+03	7.13E+03	8.81E+03	7.13E+03	6.08E+03	5.98E+03	7.54E+03	7.28E+03	7.31E+03	**5.42E+03**
F21	30	STD	2.86E+01	5.02E+01	3.15E+01	6.05E+01	5.46E+01	4.85E+01	3.81E+01	7.69E+01	1.94E+01	**1.70E+01**
		AVG	2.74E+03	2.48E+03	2.69E+03	2.55E+03	2.57E+03	2.49E+03	2.68E+03	2.65E+03	2.40E+03	**2.35E+03**
	50	STD	4.87E+01	9.43E+01	5.22E+01	9.76E+01	9.01E+01	7.08E+01	6.42E+01	9.63E+01	**2.64E+01**	2.98E+01
		AVG	3.20E+03	2.76E+03	3.09E+03	2.90E+03	2.93E+03	2.75E+03	3.10E+03	3.10E+03	2.52E+03	**2.41E+03**
	100	STD	1.04E+02	1.83E+02	1.32E+02	1.47E+02	2.59E+02	1.53E+02	1.79E+02	1.63E+02	8.70E+01	**7.18E+01**
		AVG	4.79E+03	3.73E+03	4.57E+03	4.05E+03	4.43E+03	3.78E+03	4.73E+03	4.45E+03	3.09E+03	**2.81E+03**
F22	30	STD	6.70E+02	2.59E+03	1.91E+03	2.85E+03	**1.32E+03**	1.66E+03	9.34E+02	1.54E+03	1.73E+03	1.37E+03
		AVG	8.86E+03	4.14E+03	8.91E+03	5.57E+03	7.50E+03	5.79E+03	9.18E+03	8.24E+03	4.39E+03	**2.75E+03**
	50	STD	4.38E+02	2.21E+03	**4.26E+02**	2.18E+03	1.16E+03	1.04E+03	6.24E+02	1.05E+03	2.80E+03	9.52E+02
		AVG	1.69E+04	1.42E+04	1.81E+04	1.34E+04	1.28E+04	1.07E+04	1.64E+04	1.48E+04	1.12E+04	**9.82E+03**
	100	STD	**4.59E+02**	2.70E+03	6.08E+02	4.61E+03	1.72E+03	1.63E+03	1.01E+03	1.36E+03	2.11E+03	2.17E+03
		AVG	3.48E+04	2.96E+04	3.70E+04	2.96E+04	2.78E+04	**2.19E+04**	3.46E+04	3.18E+04	3.22E+04	2.49E+04
F23	30	STD	4.67E+01	7.65E+01	1.20E+02	7.52E+01	1.78E+02	3.49E+01	2.16E+02	1.15E+02	4.82E+01	**3.48E+01**
		AVG	3.35E+03	2.87E+03	3.39E+03	3.00E+03	3.29E+03	2.83E+03	3.54E+03	3.18E+03	2.82E+03	**2.73E+03**
	50	STD	1.08E+02	1.40E+02	1.31E+02	1.54E+02	2.25E+02	**6.31E+01**	2.57E+02	1.40E+02	7.71E+01	7.72E+01
		AVG	4.13E+03	3.35E+03	4.23E+03	3.57E+03	4.03E+03	3.18E+03	4.58E+03	3.84E+03	3.10E+03	**2.93E+03**
	100	STD	1.70E+02	1.96E+02	4.38E+02	2.28E+02	3.82E+02	1.13E+02	4.69E+02	2.80E+02	8.28E+01	**7.78E+01**
		AVG	6.14E+03	4.31E+03	6.63E+03	4.74E+03	5.84E+03	3.90E+03	7.47E+03	5.36E+03	3.72E+03	**3.29E+03**
F24	30	STD	8.69E+01	6.71E+01	8.50E+01	1.08E+02	1.39E+02	**3.57E+01**	1.48E+02	9.51E+01	3.81E+01	3.84E+01
		AVG	3.64E+03	3.03E+03	3.57E+03	3.18E+03	3.57E+03	2.99E+03	3.85E+03	3.27E+03	2.96E+03	**2.91E+03**
	50	STD	1.87E+02	1.48E+02	1.45E+02	1.93E+02	2.54E+02	6.07E+01	2.68E+02	1.64E+02	**5.37E+01**	5.82E+01
		AVG	4.36E+02	3.28E+02	7.34E+02	4.36E+02	6.86E+02	**1.61E+02**	1.10E+03	4.78E+02	2.18E+02	2.04E+02
	100	STD	4.36E+02	3.28E+02	7.34E+02	4.36E+02	6.86E+02	**1.61E+02**	1.10E+03	4.78E+02	2.18E+02	2.04E+02
		AVG	9.27E+03	5.36E+03	1.06E+04	6.28E+03	8.46E+03	4.50E+03	1.18E+04	6.65E+03	4.80E+03	**4.16E+03**
F25	30	STD	1.63E+02	3.75E+01	4.42E+02	5.93E+01	4.43E+01	3.72E+02	8.88E+02	6.06E+01	2.26E+01	**1.56E+01**
		AVG	4.46E+03	2.97E+03	4.77E+03	2.98E+03	3.02E+03	3.29E+03	5.63E+03	3.22E+03	2.93E+03	**2.90E+03**
	50	STD	8.69E+02	3.27E+02	1.69E+03	2.69E+03	2.52E+02	2.53E+03	2.05E+03	3.98E+02	1.32E+02	**2.83E+01**
		AVG	1.46E+04	3.88E+03	1.40E+04	4.26E+03	3.85E+03	6.40E+03	1.57E+04	5.12E+03	3.35E+03	**3.13E+03**
	100	STD	1.03E+03	1.42E+03	2.96E+03	6.42E+03	5.91E+02	7.63E+03	3.21E+03	1.14E+03	4.58E+02	**8.04E+01**
		AVG	2.77E+04	8.17E+03	3.04E+04	1.00E+04	6.82E+03	1.99E+04	2.90E+04	1.06E+04	5.58E+03	**3.81E+03**
F26	30	STD	7.20E+02	1.18E+03	5.63E+02	1.05E+03	1.15E+03	4.01E+02	1.00E+03	1.13E+03	4.41E+02	**2.53E+02**
		AVG	1.07E+04	6.29E+03	9.91E+03	7.18E+03	8.02E+03	5.75E+03	1.07E+04	9.01E+03	5.70E+03	**4.48E+03**
	50	STD	**4.60E+02**	2.03E+03	8.79E+02	2.06E+03	1.21E+03	9.44E+02	1.33E+03	1.48E+03	8.71E+02	7.89E+02
		AVG	1.68E+04	1.20E+04	1.71E+04	1.07E+04	1.18E+04	8.79E+03	1.70E+04	1.52E+04	8.10E+03	**6.19E+03**
	100	STD	**1.14E+03**	2.61E+03	3.39E+03	4.53E+03	1.80E+03	1.75E+03	4.21E+03	3.44E+03	1.49E+03	1.64E+03
		AVG	5.12E+04	3.27E+04	5.04E+04	2.70E+04	3.12E+04	1.98E+04	5.22E+04	3.82E+04	1.98E+04	**1.38E+04**
F27	30	STD	1.31E+02	5.55E+01	1.62E+02	6.76E+01	2.56E+02	2.83E+01	3.27E+02	1.21E+02	3.30E+01	**1.40E+01**
		AVG	4.06E+03	3.28E+03	4.09E+03	3.33E+03	3.65E+03	3.26E+03	4.67E+03	3.46E+03	3.30E+03	**3.25E+03**
	50	STD	4.18E+02	2.80E+02	3.85E+02	2.48E+02	7.74E+02	1.18E+02	5.30E+02	6.56E+02	1.73E+02	**7.28E+01**
		AVG	6.24E+03	3.88E+03	6.77E+03	3.98E+03	5.13E+03	**3.62E+03**	6.98E+03	4.99E+03	3.84E+03	3.68E+03
	100	STD	6.95E+02	2.90E+02	1.16E+03	5.00E+02	9.64E+02	2.19E+02	1.42E+03	1.13E+03	1.89E+02	**1.31E+02**
		AVG	1.26E+04	4.52E+03	1.15E+04	4.74E+03	6.93E+03	4.03E+03	1.35E+04	6.26E+03	4.33E+03	**4.03E+03**
F28	30	STD	2.85E+02	6.05E+01	6.14E+02	7.85E+02	7.84E+01	8.90E+02	1.18E+03	2.36E+02	5.26E+01	**5.87E+00**
		AVG	6.44E+03	3.38E+03	6.13E+03	3.68E+03	3.49E+03	4.13E+03	6.89E+03	3.82E+03	3.37E+03	**3.28E+03**
	50	STD	5.42E+02	3.70E+02	1.08E+03	2.43E+03	3.78E+02	8.98E+02	1.18E+03	5.10E+02	4.88E+02	**6.80E+01**
		AVG	1.24E+04	4.42E+03	1.17E+04	6.53E+03	5.03E+03	8.51E+03	1.23E+04	6.03E+03	4.25E+03	**3.54E+03**
	100	STD	8.37E+02	1.33E+03	2.64E+03	6.05E+03	8.38E+02	3.24E+03	3.38E+03	1.18E+03	1.71E+03	**2.80E+02**
		AVG	2.75E+04	1.10E+04	3.16E+04	1.77E+04	9.52E+03	2.09E+04	3.43E+04	1.48E+04	9.96E+03	**4.52E+03**
F29	30	STD	6.61E+02	2.54E+02	5.15E+02	3.93E+02	4.78E+02	3.20E+02	2.15E+03	4.91E+02	2.85E+02	**1.26E+02**
		AVG	7.34E+03	4.15E+03	6.36E+03	4.52E+03	5.01E+03	4.20E+03	7.69E+03	5.44E+03	4.06E+03	**3.69E+03**
	50	STD	1.78E+04	7.41E+02	6.25E+03	1.25E+03	9.20E+02	5.51E+02	7.48E+04	3.08E+03	3.67E+02	**3.09E+02**
		AVG	3.29E+04	5.84E+03	1.67E+04	6.36E+03	7.22E+03	5.20E+03	7.07E+04	1.04E+04	5.13E+03	**4.42E+03**
	100	STD	1.78E+05	1.32E+03	1.77E+05	3.22E+03	1.53E+03	3.58E+03	6.14E+05	3.77E+03	8.23E+02	**4.96E+02**
		AVG	4.96E+05	1.12E+04	2.01E+05	1.26E+04	1.35E+04	1.13E+04	8.45E+05	2.09E+04	9.23E+03	**8.07E+03**
F30	30	STD	4.73E+08	1.09E+06	1.90E+08	2.10E+06	2.64E+07	8.94E+05	1.25E+09	5.08E+07	3.75E+05	**2.37E+05**
		AVG	1.22E+09	1.04E+06	4.11E+08	1.51E+06	1.68E+07	6.71E+05	2.15E+09	6.43E+07	**2.26E+05**	3.44E+05
	50	STD	1.16E+09	1.29E+07	1.34E+09	5.86E+07	6.16E+07	5.97E+07	3.02E+09	1.00E+08	9.04E+06	**5.31E+06**
		AVG	4.73E+09	3.29E+07	3.30E+09	5.00E+07	1.27E+08	3.08E+07	7.72E+09	3.04E+08	**1.45E+07**	1.81E+07
	100	STD	4.35E+09	1.25E+09	5.03E+09	1.85E+08	3.49E+08	2.28E+09	7.59E+09	1.37E+09	1.45E+07	**7.56E+06**
		AVG	3.96E+10	7.34E+08	2.55E+10	3.00E+08	7.08E+08	2.90E+09	4.10E+10	2.90E+09	**2.18E+07**	2.33E+07

**Table 4:** Friedman test of the MSO algorithm compared to other night algorithms on CEC 2017.

**Dim**	**BWO**	**COA**	**CPO**	**DBO**	**HHO**	**MFO**	**AOA**	**WOA**	**SO**	**MSO**
30	9	3.833	8.7	4.566	5.566	4.233	8.733	7.133	1.966	**1.2**
50	8.866	4.066	8.8	4.766	4.933	4.433	8.9	6.933	2.133	**1.166**
100	8.8	4.1	8.9	4.8	4.366	5	8.6	6.566	2.733	**1.133**
Friedman Value	8.88	3.99	8.8	4.705	4.955	4.555	8.744	6.877	2.277	**1.166**
Ranking	10	3	9	5	6	4	8	7	2	**1**

**Table 5:** Wilcoxon rank sum test (Dim = 30).

	BWO	COA	CPO	DBO	HHO	MFO	AOA	WOA	SO
F1	3.02E−11 < 0.05	3.02E−11 < 0.05	3.02E−11 < 0.05	3.02E−11 < 0.05	3.02E−11 < 0.05	3.02E−11 < 0.05	3.02E−11 < 0.05	3.02E−11 < 0.05	3.02E−11 < 0.05
F2	3.02E−11 < 0.05	5.46E−09 < 0.05	3.02E−11 < 0.05	3.02E−11 < 0.05	3.02E−11 < 0.05	3.02E−11 < 0.05	3.02E−11 < 0.05	3.02E−11 < 0.05	5.49E−11 < 0.05
F3	3.65E−08 < 0.05	3.47E−10 < 0.05	3.02E−11 < 0.05	1.21E−10 < 0.05	4.92E−01 < 0.05	1.61E−10 < 0.05	7.38E−10 < 0.05	3.02E−11 < 0.05	3.18E−04 < 0.05
F4	3.02E−11 < 0.05	3.69E−11 < 0.05	3.02E−11 < 0.05	2.37E−10 < 0.05	3.02E−11 < 0.05	4.50E−11 < 0.05	3.02E−11 < 0.05	3.02E−11 < 0.05	4.80E−07 < 0.05
F5	3.02E−11 < 0.05	3.02E−11 < 0.05	3.02E−11 < 0.05	3.02E−11 < 0.05	3.02E−11 < 0.05	3.34E−11 < 0.05	3.02E−11 < 0.05	3.02E−11 < 0.05	1.20E−08 < 0.05
F6	3.02E−11 < 0.05	3.02E−11 < 0.05	3.02E−11 < 0.05	3.02E−11 < 0.05	3.02E−11 < 0.05	3.02E−11 < 0.05	3.02E−11 < 0.05	3.02E−11 < 0.05	8.99E−11 < 0.05
F7	3.02E−11 < 0.05	3.02E−11 < 0.05	3.02E−11 < 0.05	3.34E−11 < 0.05	3.02E−11 < 0.05	3.02E−11 < 0.05	3.02E−11 < 0.05	3.02E−11 < 0.05	3.69E−11 < 0.05
F8	3.02E−11 < 0.05	3.02E−11 < 0.05	3.02E−11 < 0.05	3.02E−11 < 0.05	3.02E−11 < 0.05	3.02E−11 < 0.05	3.02E−11 < 0.05	3.02E−11 < 0.05	1.46E−10 < 0.05
F9	3.02E−11 < 0.05	3.02E−11 < 0.05	3.02E−11 < 0.05	3.02E−11 < 0.05	3.02E−11 < 0.05	3.02E−11 < 0.05	3.02E−11 < 0.05	3.02E−11 < 0.05	6.52E−09 < 0.05
F10	3.02E−11 < 0.05	4.44E−07 < 0.05	3.02E−11 < 0.05	4.31E−08 < 0.05	4.57E−09 < 0.05	2.05E−03 < 0.05	3.02E−11 < 0.05	8.99E−11 < 0.05	2.01E−04 < 0.05
F11	3.02E−11 < 0.05	3.02E−11 < 0.05	3.02E−11 < 0.05	3.02E−11 < 0.05	3.02E−11 < 0.05	3.02E−11 < 0.05	3.02E−11 < 0.05	3.02E−11 < 0.05	6.70E−11 < 0.05
F12	3.02E−11 < 0.05	2.15E−06 < 0.05	3.02E−11 < 0.05	5.53E−08 < 0.05	4.50E−11 < 0.05	3.65E−08 < 0.05	3.02E−11 < 0.05	3.02E−11 < 0.05	2.71E−01
F13	3.02E−11 < 0.05	5.27E−05 < 0.05	3.02E−11 < 0.05	1.16E−07 < 0.05	3.02E−11 < 0.05	2.68E−04 < 0.05	3.02E−11 < 0.05	3.02E−11 < 0.05	2.88E−06 < 0.05
F14	3.02E−11 < 0.05	3.01E−07 < 0.05	3.02E−11 < 0.05	1.85E−08 < 0.05	1.46E−10 < 0.05	8.89E−10 < 0.05	3.02E−11 < 0.05	6.07E−11 < 0.05	1.54E−01
F15	3.02E−11 < 0.05	5.79E−01 < 0.05	3.02E−11 < 0.05	1.25E−07 < 0.05	4.98E−11 < 0.05	1.52E−03 < 0.05	4.11E−07 < 0.05	3.02E−11 < 0.05	8.68E−03 < 0.05
F16	3.02E−11 < 0.05	2.92E−09 < 0.05	3.02E−11 < 0.05	2.87E−10 < 0.05	8.99E−11 < 0.05	5.97E−09 < 0.05	3.02E−11 < 0.05	3.02E−11 < 0.05	3.92E−02 < 0.05
F17	3.02E−11 < 0.05	1.09E−10 < 0.05	3.02E−11 < 0.05	3.02E−11 < 0.05	3.34E−11 < 0.05	6.70E−11 < 0.05	3.02E−11 < 0.05	3.02E−11 < 0.05	3.20E−09 < 0.05
F18	3.02E−11 < 0.05	4.12E−06 < 0.05	3.02E−11 < 0.05	1.64E−05 < 0.05	1.31E−08 < 0.05	9.76E−10 < 0.05	3.34E−11 < 0.05	1.21E−10 < 0.05	2.39E−04 < 0.05
F19	3.02E−11 < 0.05	7.62E−01 < 0.05	3.02E−11 < 0.05	2.15E−06 < 0.05	3.02E−11 < 0.05	6.28E−06 < 0.05	3.02E−11 < 0.05	3.02E−11 < 0.05	5.11E−01
F20	3.02E−11 < 0.05	1.01E−08 < 0.05	3.02E−11 < 0.05	1.61E−10 < 0.05	6.07E−11 < 0.05	3.50E−09 < 0.05	1.09E−10 < 0.05	4.98E−11 < 0.05	4.35E−05 < 0.05
F21	3.02E−11 < 0.05	3.69E−11 < 0.05	3.02E−11 < 0.05	3.34E−11 < 0.05	3.02E−11 < 0.05	3.34E−11 < 0.05	3.02E−11 < 0.05	3.02E−11 < 0.05	1.17E−09 < 0.05
F22	4.08E−11 < 0.05	6.53E−08 < 0.05	1.61E−10 < 0.05	9.26E−09 < 0.05	3.82E−10 < 0.05	8.48E−09 < 0.05	4.08E−11 < 0.05	9.92E−11 < 0.05	9.06E−08 < 0.05
F23	3.02E−11 < 0.05	1.61E−10 < 0.05	3.02E−11 < 0.05	3.02E−11 < 0.05	3.02E−11 < 0.05	4.20E−10 < 0.05	3.02E−11 < 0.05	3.02E−11 < 0.05	4.57E−09 < 0.05
F24	3.02E−11 < 0.05	4.20E−10 < 0.05	3.02E−11 < 0.05	3.69E−11 < 0.05	3.02E−11 < 0.05	1.86E−09 < 0.05	3.02E−11 < 0.05	3.02E−11 < 0.05	4.74E−06 < 0.05
F25	3.02E−11 < 0.05	1.33E−10 < 0.05	3.02E−11 < 0.05	8.10E−10 < 0.05	3.02E−11 < 0.05	7.38E−10 < 0.05	3.02E−11 < 0.05	3.02E−11 < 0.05	8.84E−07 < 0.05
F26	3.02E−11 < 0.05	4.18E−09 < 0.05	3.02E−11 < 0.05	5.57E−10 < 0.05	2.37E−10 < 0.05	3.02E−11 < 0.05	3.02E−11 < 0.05	3.02E−11 < 0.05	1.78E−10 < 0.05
F27	3.02E−11 < 0.05	1.49E−04 < 0.05	3.02E−11 < 0.05	3.20E−09 < 0.05	3.02E−11 < 0.05	1.02E−01	3.02E−11 < 0.05	3.34E−11 < 0.05	2.23E−09 < 0.05
F28	3.02E−11 < 0.05	5.49E−11 < 0.05	3.02E−11 < 0.05	5.57E−10 < 0.05	3.02E−11 < 0.05	3.02E−11 < 0.05	3.02E−11 < 0.05	3.02E−11 < 0.05	5.57E−10 < 0.05
F29	3.02E−11 < 0.05	2.44E−09 < 0.05	3.02E−11 < 0.05	4.98E−11 < 0.05	3.02E−11 < 0.05	1.01E−08 < 0.05	3.02E−11 < 0.05	3.02E−11 < 0.05	8.84E−07 < 0.05
F30	3.02E−11 < 0.05	6.91E−04 < 0.05	3.02E−11 < 0.05	8.77E−02	3.02E−11 < 0.05	7.51E−01	3.02E−11 < 0.05	3.02E−11 < 0.05	6.91E−04 < 0.05

**Table 6:** Wilcoxon rank test (Dim = 50).

	BWO	COA	CPO	DBO	HHO	MFO	AOA	WOA	SO
F1	3.02E−11 < 0.05	3.02E−11 < 0.05	3.02E−11 < 0.05	3.02E−11 < 0.05	3.02E−11 < 0.05	3.02E−11 < 0.05	3.02E−11 < 0.05	3.02E−11 < 0.05	3.02E−11 < 0.05
F2	3.02E−11 < 0.05	3.02E−11 < 0.05	3.02E−11 < 0.05	3.02E−11 < 0.05	3.02E−11 < 0.05	3.02E−11 < 0.05	3.02E−11 < 0.05	3.02E−11 < 0.05	3.02E−11 < 0.05
F3	1.33E−10 < 0.05	3.02E−11 < 0.05	3.02E−11 < 0.05	5.57E−10 < 0.05	1.11E−03 < 0.05	3.02E−11 < 0.05	3.83E−05 < 0.05	4.20E−10 < 0.05	2.97E−01 < 0.05
F4	3.02E−11 < 0.05	3.02E−11 < 0.05	3.02E−11 < 0.05	3.02E−11 < 0.05	3.02E−11 < 0.05	3.02E−11 < 0.05	3.02E−11 < 0.05	3.02E−11 < 0.05	2.37E−10 < 0.05
F5	3.02E−11 < 0.05	3.02E−11 < 0.05	3.02E−11 < 0.05	3.02E−11 < 0.05	3.02E−11 < 0.05	3.02E−11 < 0.05	3.02E−11 < 0.05	3.02E−11 < 0.05	2.92E−09 < 0.05
F6	3.02E−11 < 0.05	3.02E−11 < 0.05	3.02E−11 < 0.05	3.02E−11 < 0.05	3.02E−11 < 0.05	3.02E−11 < 0.05	3.02E−11 < 0.05	3.02E−11 < 0.05	3.47E−10 < 0.05
F7	3.02E−11 < 0.05	3.02E−11 < 0.05	3.02E−11 < 0.05	3.02E−11 < 0.05	3.02E−11 < 0.05	3.02E−11 < 0.05	3.02E−11 < 0.05	3.02E−11 < 0.05	3.34E−11 < 0.05
F8	3.02E−11 < 0.05	3.02E−11 < 0.05	3.02E−11 < 0.05	3.02E−11 < 0.05	3.02E−11 < 0.05	3.02E−11 < 0.05	3.02E−11 < 0.05	3.02E−11 < 0.05	3.02E−11 < 0.05
F9	3.02E−11 < 0.05	3.02E−11 < 0.05	3.02E−11 < 0.05	3.02E−11 < 0.05	3.02E−11 < 0.05	3.02E−11 < 0.05	3.02E−11 < 0.05	3.02E−11 < 0.05	4.20E−10 < 0.05
F10	3.02E−11 < 0.05	3.69E−11 < 0.05	3.02E−11 < 0.05	1.73E−07 < 0.05	3.35E−08 < 0.05	1.58E−01	3.02E−11 < 0.05	3.02E−11 < 0.05	4.84E−02 < 0.05
F11	3.02E−11 < 0.05	1.09E−10 < 0.05	3.02E−11 < 0.05	1.69E−09 < 0.05	1.43E−08 < 0.05	3.34E−11 < 0.05	3.02E−11 < 0.05	3.02E−11 < 0.05	1.20E−08 < 0.05
F12	3.02E−11 < 0.05	3.69E−11 < 0.05	3.02E−11 < 0.05	3.02E−11 < 0.05	3.02E−11 < 0.05	3.02E11 < 0.05	3.02E−11 < 0.05	3.02E−11 < 0.05	1.17E−04 < 0.05
F13	3.02E−11 < 0.05	4.98E−11 < 0.05	3.02E−11 < 0.05	3.02E−11 < 0.05	3.02E−11 < 0.05	2.60E−08 < 0.05	3.02E−11 < 0.05	3.02E−11 < 0.05	1.04E−04 < 0.05
F14	3.02E−11 < 0.05	5.86E−06 < 0.05	3.02E−11 < 0.05	5.46E−09 < 0.05	4.98E−11 < 0.05	5.46E−09 < 0.05	3.02E−11 < 0.05	3.34E−11 < 0.05	3.99E−04 < 0.05
F15	3.02E−11 < 0.05	1.86E−06 < 0.05	3.02E−11 < 0.05	2.39E−08 < 0.05	3.02E−11 < 0.05	3.32E−06 < 0.05	3.02E−11 < 0.05	3.02E−11 < 0.05	8.65E−01
F16	3.02E−11 < 0.05	5.00E−09 < 0.05	3.02E−11 < 0.05	7.39E−11 < 0.05	6.70E−11 < 0.05	5.57E−10 < 0.05	3.02E−11 < 0.05	3.02E−11 < 0.05	1.49E−04 < 0.05
F17	3.02E−11 < 0.05	3.20E−09 < 0.05	3.02E−11 < 0.05	3.02E−11 < 0.05	2.61E−10 < 0.05	7.39E−11 < 0.05	3.02E−11 < 0.05	3.02E−11 < 0.05	1.03E−06 < 0.05
F18	3.02E−11 < 0.05	4.94E−05 < 0.05	3.02E−11 < 0.05	2.60E−05 < 0.05	1.47E−07 < 0.05	1.61E−06 < 0.05	3.02E−11 < 0.05	4.08E−11 < 0.05	5.75E−02
F19	3.02E−11 < 0.05	4.11E−07 < 0.05	3.02E−11 < 0.05	5.07E−10 < 0.05	3.02E−11 < 0.05	4.80E−07 < 0.05	3.02E−11 < 0.05	3.02E−11 < 0.05	7.98E−02
F20	3.02E−11 < 0.05	3.20E−09 < 0.05	3.02E−11 < 0.05	5.00E−09 < 0.05	1.60E−07 < 0.05	6.53E−08 < 0.05	1.09E−10 < 0.05	3.69E−11 < 0.05	1.37E−01
F21	3.02E−11 < 0.05	3.02E−11 < 0.05	3.02E−11 < 0.05	3.02E−11 < 0.05	3.02E−11 < 0.05	3.02E−11 < 0.05	3.02E−11 < 0.05	3.02E−11 < 0.05	4.50E−11 < 0.05
F22	3.02E−11 < 0.05	1.55E−09 < 0.05	3.02E−11 < 0.05	7.12E−09 < 0.05	2.37E−10 < 0.05	1.17E−03 < 0.05	3.02E−11 < 0.05	3.02E−11 < 0.05	7.98E−02
F23	3.02E−11 < 0.05	4.08E−11 < 0.05	3.02E−11 < 0.05	3.02E−11 < 0.05	3.02E−11 < 0.05	1.21E−10 < 0.05	3.02E−11 < 0.05	3.02E−11 < 0.05	6.52E−09 < 0.05
F24	3.02E−11 < 0.05	3.34E−11 < 0.05	3.02E−11 < 0.05	3.02E−11 < 0.05	3.02E−11 < 0.05	1.46E−10 < 0.05	3.02E−11 < 0.05	3.02E−11 < 0.05	1.55E−09 < 0.05
F25	3.02E−11 < 0.05	3.02E−11 < 0.05	3.02E−11 < 0.05	3.02E−11 < 0.05	3.02E−11 < 0.05	3.02E−11 < 0.05	3.02E−11 < 0.05	3.02E−11 < 0.05	4.08E−11 < 0.05
F26	3.02E−11 < 0.05	3.82E−10 < 0.05	3.02E−11 < 0.05	9.26E−09 < 0.05	3.34E−11 < 0.05	2.37E−10 < 0.05	3.02E−11 < 0.05	3.02E−11 < 0.05	2.23E−09 < 0.05
F27	3.02E−11 < 0.05	1.17E−04 < 0.05	3.02E−11 < 0.05	2.39E−08 < 0.05	3.02E−11 < 0.05	1.76E−02 < 0.05	3.02E−11 < 0.05	3.02E−11 < 0.05	5.27E−05 < 0.05
F28	3.02E−11 < 0.05	3.02E−11 < 0.05	3.02E−11 < 0.05	4.50E−11 < 0.05	3.02E−11 < 0.05	3.02E−11 < 0.05	3.02E−11 < 0.05	3.02E−11 < 0.05	5.07E−10 < 0.05
F29	3.02E−11 < 0.05	9.92E−11 < 0.05	3.02E−11 < 0.05	1.96E−10 < 0.05	3.02E−11 < 0.05	2.03E−07 < 0.05	3.02E−11 < 0.05	3.02E−11 < 0.05	1.20E−08 < 0.05
F30	3.02E−11 < 0.05	2.88E−06 < 0.05	3.02E−11 < 0.05	9.52E−04 < 0.05	3.02E−11 < 0.05	2.92E−02 < 0.05	3.02E−11 < 0.05	3.02E−01 < 0.05	2.38E−03 < 0.05

**Table 7:** Wilcoxon rank test (Dim = 100).

	BWO	COA	CPO	DBO	HHO	MFO	AOA	WOA	SO
F1	3.02E−11 < 0.05	3.02E−11 < 0.05	3.02E−11 < 0.05	3.02E−11 < 0.05	3.02E−11 < 0.05	3.02E−11 < 0.05	3.02E−11 < 0.05	3.02E−11 < 0.05	3.02E−11 < 0.05
F2	3.02E−11 < 0.05	3.02E−11 < 0.05	3.02E−11 < 0.05	3.02E−11 < 0.05	3.02E−11 < 0.05	3.02E−11 < 0.05	3.02E−11 < 0.05	3.02E−11 < 0.05	3.34E−11 < 0.05
F3	1.68E−03 < 0.05	3.02E−11 < 0.05	3.02E−11 < 0.05	9.26E−09 < 0.05	1.19E−01	3.02E−11 < 0.05	1.22E−01	3.02E−11 < 0.05	1.38E−02 < 0.05
F4	3.02E−11 < 0.05	3.02E−11 < 0.05	3.02E−11 < 0.05	3.02E−11 < 0.05	3.02E−11 < 0.05	3.02E−11 < 0.05	3.02E−11 < 0.05	3.02E−11 < 0.05	3.02E−11 < 0.05
F5	3.02E−11 < 0.05	3.02E−11 < 0.05	3.02E−11 < 0.05	3.02E−11 < 0.05	3.02E−11 < 0.05	3.02E−11 < 0.05	3.02E−11 < 0.05	3.02E−11 < 0.05	3.69E−11 < 0.05
F6	3.02E−11 < 0.05	3.02E−11 < 0.05	3.02E−11 < 0.05	3.02E−11 < 0.05	3.02E−11 < 0.05	3.02E−11 < 0.05	3.02E−11 < 0.05	3.02E−11 < 0.05	3.34E−11 < 0.05
F7	3.02E−11 < 0.05	3.02E−11 < 0.05	3.02E−11 < 0.05	3.02E−11 < 0.05	3.02E−11 < 0.05	3.02E−11 < 0.05	3.02E−11 < 0.05	3.02E−11 < 0.05	3.02E−11 < 0.05
F8	3.02E−11 < 0.05	3.02E−11 < 0.05	3.02E−11 < 0.05	3.02E−11 < 0.05	3.02E−11 < 0.05	3.02E−11 < 0.05	3.02E−11 < 0.05	3.02E−11 < 0.05	4.50E−11 < 0.05
F9	3.02E−11 < 0.05	3.02E−11 < 0.05	3.02E−11 < 0.05	3.02E−11 < 0.05	3.02E−11 < 0.05	3.02E−11 < 0.05	3.02E−11 < 0.05	3.02E−11 < 0.05	6.05E−07 < 0.05
F10	3.02E−11 < 0.05	6.31E−01	3.02E−11 < 0.05	1.68E−04 < 0.05	7.30E−04 < 0.05	7.09E−08 < 0.05	3.02E−11 < 0.05	8.99E−11 < 0.05	3.02E−11 < 0.05
F11	3.02E−11 < 0.05	3.02E−11 < 0.05	3.02E−11 < 0.05	3.02E−11 < 0.05	1.10E−08 < 0.05	8.15E−11 < 0.05	3.34E−11 < 0.05	3.34E−11 < 0.05	7.38E−10 < 0.05
F12	3.02E−11 < 0.05	3.02E−11 < 0.05	3.02E−11 < 0.05	3.02E−11 < 0.05	3.02E−11 < 0.05	3.02E−11 < 0.05	3.02E−11 < 0.05	3.02E−11 < 0.05	3.02E−11 < 0.05
F13	3.02E−11 < 0.05	3.02E−11 < 0.05	3.02E−11 < 0.05	3.02E−11 < 0.05	3.02E−11 < 0.05	3.02E−11 < 0.05	3.02E−11 < 0.05	3.02E−11 < 0.05	3.02E−11 < 0.05
F14	3.02E−11 < 0.05	2.78E−07 < 0.05	3.02E−11 < 0.05	1.09E−10 < 0.05	6.70E−11 < 0.05	2.23E−09 < 0.05	3.02E−11 < 0.05	3.34E−11 < 0.05	4.44E−07 < 0.05
F15	3.02E−11 < 0.05	3.02E−11 < 0.05	3.02E−11 < 0.05	3.02E−11 < 0.05	3.02E−11 < 0.05	3.02E−11 < 0.05	3.02E−11 < 0.05	3.02E−11 < 0.05	1.16E−07 < 0.05
F16	3.02E−11 < 0.05	1.09E−10 < 0.05	3.02E−11 < 0.05	4.98E−11 < 0.05	3.34E−11 < 0.05	1.33E−10 < 0.05	3.02E−11 < 0.05	3.02E−11 < 0.05	4.98E−04 < 0.05
F17	3.02E−11 < 0.05	3.47E−10 < 0.05	3.02E−11 < 0.05	3.02E−11 < 0.05	3.02E−11 < 0.05	4.08E−11 < 0.05	3.02E−11 < 0.05	3.02E−11 < 0.05	4.31E−08 < 0.05
F18	3.02E−11 < 0.05	9.79E−05 < 0.05	3.02E−11 < 0.05	8.15E−11 < 0.05	1.73E−06 < 0.05	5.57E−10 < 0.05	3.02E−11 < 0.05	5.49E−11 < 0.05	2.60E−08 < 0.05
F19	3.02E−11 < 0.05	9.92E−11 < 0.05	3.02E−11 < 0.05	7.39E−11 < 0.05	3.02E−11 < 0.05	4.50E−11 < 0.05	3.02E−11 < 0.05	3.02E−11 < 0.05	6.31E−01
F20	3.02E−11 < 0.05	3.34E−11 < 0.05	3.02E−11 < 0.05	1.33E−10 < 0.05	1.87E−05 < 0.05	1.11E−03 < 0.05	4.08E−11 < 0.05	4.98E−11 < 0.05	4.98E−11 < 0.05
F21	3.02E−11 < 0.05	3.02E−11 < 0.05	3.02E−11 < 0.05	3.02E−11 < 0.05	3.02E−11 < 0.05	3.02E−11 < 0.05	3.02E−11 < 0.05	3.02E−11 < 0.05	6.07E−11 < 0.05
F22	3.02E−11 < 0.05	3.65E−08 < 0.05	3.02E−11 < 0.05	4.94E−05 < 0.05	2.68E−06 < 0.05	7.60E−07 < 0.05	3.02E−11 < 0.05	3.69E−11 < 0.05	8.15E−11 < 0.05
F23	3.02E−11 < 0.05	3.02E−11 < 0.05	3.02E−11 < 0.05	3.02E−11 < 0.05	3.02E−11 < 0.05	3.02E−11 < 0.05	3.02E−11 < 0.05	3.02E−11 < 0.05	3.02E−11 < 0.05
F24	3.02E−11 < 0.05	3.02E−11 < 0.05	3.02E−11 < 0.05	3.02E−11 < 0.05	3.02E−11 < 0.05	9.83E−08 < 0.05	3.02E−11 < 0.05	3.02E−11 < 0.05	1.21E−10 < 0.05
F25	3.02E−11 < 0.05	3.02E−11 < 0.05	3.02E−11 < 0.05	3.02E−11 < 0.05	3.02E−11 < 0.05	3.02E−11 < 0.05	3.02E−11 < 0.05	3.02E−11 < 0.05	3.02E−11 < 0.05
F26	3.02E−11 < 0.05	3.02E−11 < 0.05	3.02E−11 < 0.05	3.02E−11 < 0.05	3.02E−11 < 0.05	3.02E−11 < 0.05	3.02E−11 < 0.05	3.02E−11 < 0.05	3.02E−11 < 0.05
F27	3.02E−11 < 0.05	2.23E−09 < 0.05	3.02E−11 < 0.05	7.12E−09 < 0.05	3.02E−11 < 0.05	8.53E−01	3.02E−11 < 0.05	3.02E−11 < 0.05	3.65E−08 < 0.05
F28	3.02E−11 < 0.05	3.02E−11 < 0.05	3.02E−11 < 0.05	3.02E−11 < 0.05	3.02E−11 < 0.05	3.02E−11 < 0.05	3.02E−11 < 0.05	3.02E−11 < 0.05	3.02E−11 < 0.05
F29	3.02E−11 < 0.05	3.69E−11 < 0.05	3.02E−11 < 0.05	7.39E−11 < 0.05	3.02E−11 < 0.05	2.87E−10 < 0.05	3.02E−11 < 0.05	3.02E−11 < 0.05	3.26E−07 < 0.05
F30	3.02E−11 < 0.05	3.02E−11 < 0.05	3.02E−11 < 0.05	3.02E−11 < 0.05	3.02E−11 < 0.05	3.02E−11 < 0.05	3.02E−11 < 0.05	3.02E−11 < 0.05	1.45E−01

**Table 8:** Comparison of ablation results.

Algorithm		SO	MSO_A	MSO_B	MSO_C	MSO
F1	STD	2.73E+07	4.22E+04	8.82E+04	3.22E+05	**3.89E+04**
	AVG	1.81E+07	1.70E+05	2.79E+05	5.07E+05	**1.58E+05**
F11	STD	9.19E+01	4.21E+01	9.56E+01	4.59E+01	**4.15E+01**
	AVG	1.44E+03	1.25E+03	1.34E+03	1.24E+03	**1.23E+03**
F14	STD	1.25E+05	5.19E+04	1.56E+05	6.16E+04	**2.49E+04**
	AVG	7.90E+04	4.27E+04	1.40E+05	3.73E+04	**3.07E+04**
F17	STD	1.18E+02	1.41E+02	2.01E+02	1.32E+02	**1.08E+02**
	AVG	2.25E+03	1.91E+03	2.20E+03	1.92E+03	**1.91E+03**
F20	STD	1.85E+02	1.67E+02	1.67E+02	1.52E+02	**1.40E+02**
	AVG	2.51E+03	2.33E+03	2.47E+03	2.32E+03	**2.29E+03**
F22	STD	1.54E+03	1.65E+03	1.52E+03	2.30E+03	**1.36E+03**
	AVG	3.74E+03	3.19E+03	3.61E+03	3.32E+03	**2.83E+03**

**Table 9:** Statistical analysis of pressure vessel design.

Algorithm	Max	Min	Mean	Medium	Std	Friedman Value
BWO	8.06E+03	6.24E+03	7.11E+03	7.13E+03	4.00E+02	6
COA	7.54E+03	6.06E+03	6.70E+03	6.61E+03	4.53E+02	4.53
CPO	1.89E+04	7.22E+03	1.19E+04	1.18E+04	2.80E+03	9.37
DBO	7.33E+03	6.06E+03	6.47E+03	6.29E+03	4.02E+02	3.4
HHO	8.23E+03	6.09E+03	6.97E+03	6.90E+03	4.91E+02	5.33
MFO	7.54E+03	6.06E+03	6.70E+03	6.41E+03	5.72E+02	4.23
AOA	2.38E+04	7.71E+03	1.23E+04	1.10E+04	4.43E+03	9.33
WOA	1.23E+04	6.41E+03	8.67E+03	8.30E+03	1.60E+03	7.9
SO	7.33E+03	6.06E+03	6.37E+03	**6.27E+03**	3.47E+02	2.57
MSO	**6.37E+03**	**6.04E+03**	**6.28E+03**	6.37E+03	**1.34E+02**	**2.33**

**Table 10:** Statistical analysis of tension/compression spring design.

Algorithm	Max	Min	Mean	Medium	Std	Friedman Value
BWO	2.15E−02	1.29E−02	1.54E−02	1.36E−02	2.77E−03	6.7
COA	1.60E−02	1.27E−02	1.32E−02	1.29E−02	7.72E−04	3.53
CPO	3.98E−02	1.66E−02	2.51E−02	2.38E−02	5.99E−03	9.73
DBO	1.82E−02	1.27E−02	1.40E−02	1.31E−02	1.92E−03	4.27
HHO	1.77E−02	1.27E−02	1.40E−02	1.35E−02	1.31E−03	5.93
MFO	1.78E−02	1.27E−02	1.37E−02	1.32E−02	1.49E−03	4.53
AOA	6.96E−02	1.32E−02	2.00E−02	1.33E−02	1.39E−02	6.57
WOA	1.78E−02	1.27E−02	1.38E−02	1.34E−02	1.33E−03	5.23
SO	1.78E−02	**1.27E−02**	1.41E−02	1.33E−02	1.64E−03	5.27
MSO	**1.41E−02**	1.27E−02	**1.30E−02**	**1.28E−02**	**3.43E−04**	**3.23**

**Table 11:** Statistical analysis of hydrostatic thrust bearing design problem.

Algorithm	Max	Min	Mean	Median	Std	Friedman Value
BWO	3.46E+17	2.97E+03	2.10E+16	4.45E+03	7.17E+16	7.47
COA	4.12E+03	2.20E+03	2.85E+03	2.77E+03	4.97E+02	3.37
CPO	1.06E+17	2.86E+03	3.71E+15	5.78E+03	1.94E+16	7.6
DBO	1.16E+09	**2.05E+03**	3.87E+07	3.20E+03	2.12E+08	4.43
HHO	1.99E+21	2.65E+03	6.65E+19	1.18E+09	3.64E+20	8.37
MFO	1.14E+09	2.23E+03	3.79E+07	3.75E+03	2.08E+08	5.57
AOA	2.06E+12	2.61E+03	3.01E+11	7.25E+03	4.91E+11	7.7
WOA	1.16E+09	2.21E+03	1.55E+08	3.97E+03	4.01E+08	6.37
SO	2.95E+03	2.06E+03	2.47E+03	2.53E+03	2.80E+02	2.3
MSO	**2.82E+03**	2.06E+03	**2.41E+03**	**2.45E+03**	**2.18E+02**	**1.83**

**Table 12:** Statistical analysis of three-dimensional UAV path planning results (case 1).

Algorithm	Max	Min	Mean	Median	Std	Friedman Value
BWO	1.03E+04	7.16E+03	8.80E+03	8.80E+03	7.77E+02	9
COA	8.30E+03	5.14E+03	5.94E+03	5.85E+03	6.97E+02	4.4
CPO	1.11E+04	8.08E+03	9.58E+03	9.61E+03	7.92E+02	9.73
DBO	6.90E+03	**5.03E+03**	5.76E+03	5.66E+03	5.20E+02	3.7
HHO	8.20E+03	5.28E+03	6.69E+03	6.65E+03	8.46E+02	6.3
MFO	6.85E+03	5.01E+03	5.81E+03	5.59E+03	5.81E+02	3.77
AOA	6.06E+03	5.15E+03	5.75E+03	5.92E+03	2.90E+02	3.87
WOA	9.22E+03	6.56E+03	7.66E+03	7.54E+03	7.23E+02	7.8
SO	7.18E+03	5.19E+03	5.82E+03	5.72E+03	5.78E+02	3.7
MSO	**6.21E+03**	5.09E+03	**5.51E+03**	**5.44E+03**	**2.56E+02**	**2.73**

**Table 13:** Statistical analysis of three-dimensional UAV path planning results (case 2).

Algorithm	Max	Min	Mean	Median	Std	Friedman Value
BWO	5.67E+02	4.02E+02	5.11E+02	5.31E+02	4.30E+01	5.26
COA	5.85E+02	3.42E+02	4.78E+02	5.31E+02	8.38E+01	4.23
CPO	8.86E+02	5.01E+02	7.07E+02	7.36E+02	8.88E+01	9
DBO	5.69E+02	3.40E+02	5.13E+02	5.37E+02	7.20E+01	5.1
HHO	6.56E+02	3.46E+02	5.20E+02	5.32E+02	9.33E+01	5.4
MFO	5.99E+02	3.40E+02	5.17E+02	5.37E+02	6.31E+01	5.5
AOA	9.55E+02	5.23E+02	7.44E+02	7.44E+02	1.06E+02	9.4
WOA	7.43E+02	3.69E+02	5.43E+02	5.39E+02	1.04E+02	6.03
SO	5.37E+02	3.40E+02	4.42E+02	4.94E+02	8.41E+01	2.87
MSO	**5.33E+02**	**3.40E+02**	**4.07E+02**	**3.42E+02**	**8.38E+01**	**2.2**

## Data Availability

The original contributions presented in this study are included in this article; further inquiries can be directed to the corresponding author.
